# A Comparison of Single Molecule and Amplification Based Sequencing of Cancer Transcriptomes

**DOI:** 10.1371/journal.pone.0017305

**Published:** 2011-03-01

**Authors:** Lee T. Sam, Doron Lipson, Tal Raz, Xuhong Cao, John Thompson, Patrice M. Milos, Dan Robinson, Arul M. Chinnaiyan, Chandan Kumar-Sinha, Christopher A. Maher

**Affiliations:** 1 Michigan Center for Translational Pathology, University of Michigan, Ann Arbor, Michigan, United States of America; 2 Center for Computational Medicine and Biology, University of Michigan, Ann Arbor, Michigan, United States of America; 3 Bioinformatics Program, University of Michigan, Ann Arbor, Michigan, United States of America; 4 Helicos BioSciences Corporation, Cambridge, Massachusetts, United States of America; 5 Department of Pathology, University of Michigan, Ann Arbor, Michigan, United States of America; 6 Howard Hughes Medical Institute, Chevy Chase, Maryland, United States of America; 7 Comprehensive Cancer Center, University of Michigan, Ann Arbor, Michigan, United States of America; 8 Department of Urology, University of Michigan, Ann Arbor, Michigan, United States of America; Victor Chang Cardiac Research Institute (VCCRI), Australia

## Abstract

The second wave of next generation sequencing technologies, referred to as single-molecule sequencing (SMS), carries the promise of profiling samples directly without employing polymerase chain reaction steps used by amplification-based sequencing (AS) methods. To examine the merits of both technologies, we examine mRNA sequencing results from single-molecule and amplification-based sequencing in a set of human cancer cell lines and tissues. We observe a characteristic coverage bias towards high abundance transcripts in amplification-based sequencing. A larger fraction of AS reads cover highly expressed genes, such as those associated with translational processes and housekeeping genes, resulting in relatively lower coverage of genes at low and mid-level abundance. In contrast, the coverage of high abundance transcripts plateaus off using SMS. Consequently, SMS is able to sequence lower- abundance transcripts more thoroughly, including some that are undetected by AS methods; however, these include many more mapping artifacts. A better understanding of the technical and analytical factors introducing platform specific biases in high throughput transcriptome sequencing applications will be critical in cross platform meta-analytic studies.

## Introduction

Sequencing samples at single-molecule resolution is seen as the next step in the evolution of Next Generation Sequencing (NGS). These technologies have already produced unprecedented amounts of data at nucleotide-level resolution, and are transforming our ability to observe biological systems. NGS technology has had a particular impact in the study of transcriptomes through mRNA sequencing, or RNA-Seq. Offering a wide dynamic range and truly global view, this NGS application is quickly supplanting existing approaches for monitoring complex transcriptomes where both transcript lengths and concentrations are highly heterogeneous. The multi-faceted nature of RNA-Seq has enabled in-depth analysis of transcript abundance [1], [2], [3], alternative splicing [4], [5], [6], [7], novel transcript detection [8], biomarker discovery [9], [10], [11], pathogen detection and characterization [12], [13], [14], and gene fusion discovery [15], [16], [17].

The first wave of ‘next generation’ sequencing platforms such as those from Applied Biosystems, Illumina, Ion Torrent, and Roche/454, utilize PCR based amplification steps in sample preparation and sequencing and are thus categorized as amplification based sequencing (AS) methods. A second set of platforms, described as ‘single molecule sequencing’ (SMS) [18] by Helicos and Pacific Biosciences, eliminate the amplification steps involved in the sample preparation and sequencing process and thus profess to provide a more accurate view of the transcriptome.

AS techniques typically involve two amplification steps; the first amplification occurs during the creation of the double-stranded cDNA library from the fragmented mRNA. The cDNAs are ligated to a pair of adapter molecules, and PCR amplified. A second amplification step is carried out with the adapter-ligated single cDNA strands hybridized to primers bound to a glass or silicon substrate to produce local clusters of identical molecules using isothermal amplification or emulsion PCR. Taken together, these two steps have the potential to selectively introduce over-represented segments and genes into AS data. It has been observed that this bias exists [19], [20], [21], [22], however its effect on transcript coverage and quantification has not been thoroughly explored in complex samples with transcripts at variable concentration. The Helicos SMS protocol involves creation of single-stranded cDNA templates directly from mRNA and hybridization of these poly-adenylated templates to complementary oligomers bound to a glass slide for sequencing (**Figure S1)**.

## Results

### Assessment of SMS RNA-Seq through transcript profiling

To systematically assess the differences between the two sequencing technologies, we analyzed RNA-Seq results from amplification-based sequencing (AS) and single-molecule sequencing (SMS) across a set of twelve cancer cell lines and tissue samples. In particular, our approach attempted to discover recurrent biases that may be introduced by the amplification steps implicit in AS. Our initial dataset used to evaluate quantification performance is comprised of samples from the prostate cancer cell lines DU145, RWPE, VCaP, and LnCaP, and one prostate cancer tumor tissue with a matched adjacent normal sample. Out of our set, three samples each of VCaP and LnCaP were structured as a time course study with 0 h, 24 h, and 48 h time points.

In our analysis of the two technologies, we chose to use the preferred alignment tool for each technology in a “best vs. best” approach. AS reads were aligned with the Bowtie aligner [23] while SMS reads were aligned with IndexDP [24] (**Figure S2**). Reads aligning to known biological contaminants such as mitochondrial DNA, ribosomal RNA, and technology-specific contaminants such as adapter sequences and long oligomers, were filtered out of the data set prior to analysis.

To assess the variation between SMS and AS technologies, we adopted a simple read counting procedure similar to other RNA-Seq quantification methodologies [1], [2]. Reads from single lanes of AS and SMS technologies run in parallel, were aligned to 56,722 University of California Santa Cruz (UCSC) transcripts (version hg18). We then enumerated reads per-transcript and normalized based on the number of high quality, non-contaminant reads per sample to obtain values in reads per million (RPM). To avoid uncertainty associated with multi-mappings to gene isoforms, only single-best mapping methods were used to quantify the genes for comparison. Single best mappings were derived from AS reads by setting Bowtie to report only the single highest quality alignment per read. Single best alignments were derived from SMS reads by accepting alignments with the highest quality scores. Values from all gene transcript isoforms, as defined by UCSC, were summed to yield values in terms of alignments per million reads for each of the 29,416 genes. Coverage values in reads per kilobase per million (RPKM) were computed by summing RPKM values of the isoforms of each gene. Through a head to head comparison between AS and SMS reads of identical samples run in parallel on the two platforms, we observed a systematic over-representation of high expressing transcripts in AS as compared to SMS. This bias resulted in reduced coverage of mid- and lower-level expression genes leading to overall lower transcript detection sensitivity in AS. Reprocessing a subset of AS samples using IndexDP and repeating the analysis ruled out technical differences in read assignment as the cause of this representation bias. As the sequencing technologies and chemistries continue to advance, we expect AS platforms will overcome the limitation of low expressed transcript detection by enhanced throughput.

### Global properties of AS and SMS results

Transcriptome sequencing was carried out in parallel on AS and SMS platforms for 12 samples including 10 prostate cancer cell lines and 2 prostate cancer tissues. Overall, we generated 2.8 to 19.7 million raw AS and SMS reads in each of the 12 samples. Approximately 30–60% of these reads passed initial filtering steps and aligned to our transcriptome reference. SMS reads were produced in two separate machine runs while AS reads were produced across 6 independent machine runs. This procedure resulted in 2.1–15 million and 2.8–8 million reads for SMS and AS, respectively, which aligned to our transcriptome reference. In 10 out of the 12 samples used in the evaluation, SMS produced more alignable reads in absolute terms, with a median of 1.39x across all 12 samples. SMS results contained more reads aligning to known contaminants, ranging from 12% to 51% of total reads, with a median of 22%. The fraction of reads aligning to contaminants in AS ranged from 2.6% to 14% with a median of 4.2%. SMS read length was variable and a filtering step restricted usable reads to a length range between 24 bp and 57 bp in the first run, and 25 bp and 64 bp in our second run, yielding a read count-weighted mean length of approximately 33 bp in each of the twelve samples (**Table S1**). A median of 97% of all SMS reads had lengths between 25 bp and 47 bp across all 12 samples (**Figure S3**). AS reads were generated at a minimum length of 36 bp in each sample, although the first and last several bases were ignored to produce high quality reads at least 34 bp in length. All AS reads were considered to have a maximum of 36 bp length. Reproducibility between technical replicates of the DU145 cell line was high for both AS and SMS methods, with a Pearson correlation of *r* = .98 for both technologies (**Figure S4**). Reads from both AS and SMS were also aligned allowing for 25 maximum mappings to assess the distribution between uniquely- and multiply- mapped reads at the gene level, although only single-best mappings were used for quantification and comparison purposes. Both technologies achieved very similar unique mapping rates of 72% and 75% in AS and SMS, respectively. From this raw aligned data, we examined the relative distribution of reads across genes observed in our samples by comparing their normalized read counts. As expected, we observed broad agreement in terms of gene expression values between the technologies (**Figure S5**). However, we observed a recurrent pattern of over-representation of high-abundance transcripts by the AS methodology as compared to SMS.

### Coverage bias in amplification-sequencing

**Figure 1:**
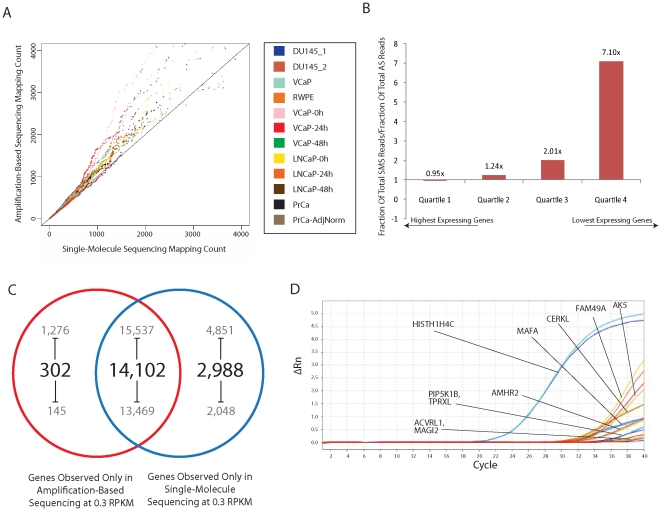
Observed bias in amplification-based sequencing. (A) Single-best mapping method-based quantile-quantile plot demonstrates evidence of over-representation of highly expressed transcripts in amplification-based sequencing compared to single-molecule methods. (B) Distribution of reads across genes by transcript concentration shows decreased SMS coverage of the most highly expressed genes, with those reads going to mid- and low-level expressors. (C) Differences in the distribution of reads lead to increased sensitivity of low-expressing transcripts. (D) Nine of the candidate genes seen above the 0.3 RPKM noise level demonstrated any amplification by RT-PCR, although only *HIST1H4C* showed high abundance.

Comparison of transcriptome reads of the same samples quantified in parallel from AS and SMS platforms reveals a distinct bias in AS results towards a slight overrepresentation of highly expressed genes as compared to SMS, as shown in **Figure 1A**. This difference was qualitatively assessed by dividing the genes into quartiles of equal number, ordered by observed values in AS, with the first quartile representing the highest expressing genes, the second quartile representing mid-level expression genes, and the third and fourth quartile defining the genes with the lowest levels of transcripts (**Figure 1B**). Highly expressed transcripts tended to have more read coverage in AS, whereas SMS tended to cover the lower expressed transcripts more effectively (**Table S3**). This additional coverage of high-concentration transcripts consistently appeared to be at the expense of lower-expressed transcripts, which tended to be more thoroughly sequenced using SMS (**Table S4**).

In order to ensure that these biases were not the result of using a different aligner for each technology, AS reads were re-aligned using the IndexDP aligner used for SMS reads for a subset of the samples, composed of the VCaP-24 h, VCaP-48 h, LnCaP-24 h, LnCaP-48 h, and DU145_1 samples (**Figure S6**). Very high correlation of gene-level values comparing Bowtie and IndexDP alignments for the set of AS reads ruled out differences between alignment tools as the source of the observed biases. For example, correlation of gene-level values in the LnCaP-24 h sample was high between alignment methods at *r*  =  0.97. Similarly high correlation levels above *r*  =  0.95 were observed in the remaining samples. Similar patterns of high-expressor over-representation in AS were observed using IndexDP alignments of AS reads in place of standard alignments using Bowtie as shown in **Figure S7**. With methodological differences essentially ruled out, we attempted to observe the effects of this high-concentration coverage bias by examining the detection of transcripts at low levels.

### Increased SMS sensitivity results from high coverage of low-abundance transcripts

To evaluate the effects of increased coverage in mid- to low- level transcripts in SMS, we calculated the number of genes observed above a noise threshold in only one of the two technologies. Using the 0.3 RPKM noise level cutoff based on Ramskold, et al. [25], the number of genes detected in only a single technology varied between a high of 4,851 and a low of 2,048 and a high of 1,276 and a low of 145 in SMS and AS (**Figure 1C**), respectively, across the set of samples. A log-fold difference between the numbers of genes detected in only one of the SMS vs. AS technology was observed as we varied the cutoff value between 0.1 RPKM and 3.0 RPKM (**Figure S8**) in 0.1 RPKM increments. These limits were chosen to examine the sensitivity of the two methods across a range of values starting from a near-zero noise level to an order of magnitude larger than previously reported. Stratification of the genes observed in a single technology into length classes of 0–300 bp, 300–3000 bp, and 3000+bp demonstrated that this was not due to differences in technology-specific sample preparation, as the AS protocol specifies a ∼300 bp size selection step that the SMS procedure does not require. This class shows relatively low representation across noise thresholds in both AS and SMS. We then took this evaluation one step further and examined the results from both SMS and AS techniques attempting to find genes detectable only in one technology.

### Uniquely detected genes in SMS

In order to substantiate potential representation biases in the two platforms and the suggested additional sensitivity of SMS, we next queried for genes which were detected above a noise threshold by SMS, but were below that threshold in AS. We chose to analyze the DU145 sample as it was the most thoroughly sequenced sample with two replicates run using each technology. Using a 0.3 RPKM threshold, we chose to test the expression of 23 genes in our DU145 samples using RT-PCR, ten of which demonstrated detectable amplification. Additionally, we sequenced the DU145 cell line much more thoroughly in order to ensure that our detections were not due to technical factors in a single machine run. As shown in **Figure S9**, this set of genes had better sequencing coverage in SMS as compared to AS across the total 94,427,789 reads generated in our second set of runs. This list was generated by examining the distribution of reads and coverage maps of the top 50 genes whose RPKM coverage showed the largest difference between AS and SMS techniques and had official HUGO names [26]. Candidates were chosen for the presence of long (>36 bp) mapping reads and well-distributed read alignments across the length of the transcripts. Of the validated genes detected only by SMS, only *HISTH1H4C* was found to be present in the DU145 sample with high confidence, as shown in **Figure 1D**. Nine other candidate genes *AK5*, *ACVRL1*, *AMHR2*, *CERKL*, *MAFA*, *MAGI2*, *PIP5K1B*, *FAM49A*, and *TPRXL* showed weak amplification. In this set of genes, amplification was only seen beyond cycle 30 making it difficult to confirm their presence. We next sought to examine the over-represented genes that may contribute to the reduction of sensitivity using amplification-based sequencing techniques.

### Consistent over-representation of high-expression genes in amplification-based sequencing

**Figure 2:**
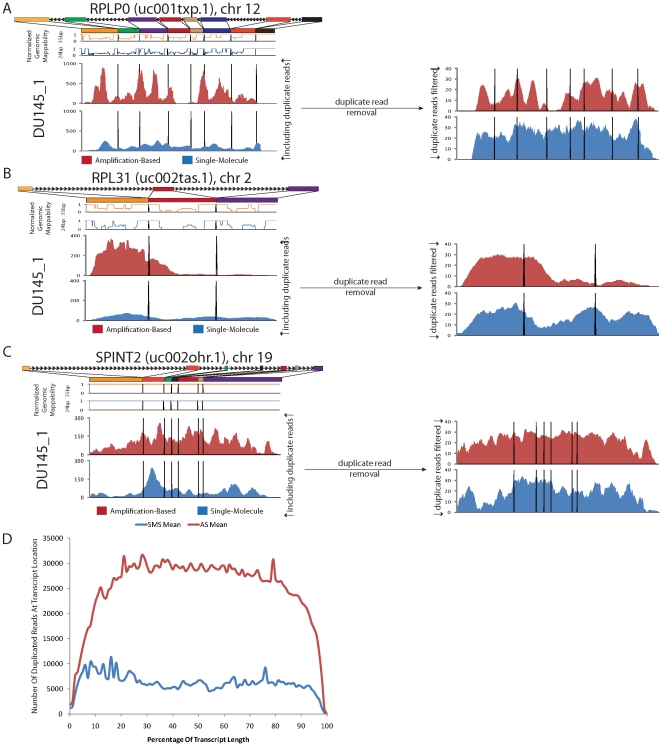
High-concentration transcript bias leads to differences in gene coverage in amplification-based sequencing. Coverage maps from amplification-based and single molecule sequencing demonstrate significantly greater coverage of (A) *RPLP0*, (B) *RPL31*, and (C) *SPINT2*. Removal of reads with the same start positions, strictly suppressing amplification of specific mRNA fragments, significantly reduces the “spikiness” seen in these cases. (D) Duplicate reads, defined as reads in excess of one per start locus and read length, are relatively evenly distributed along the length of all observed transcripts across all samples in our evaluation set.

Overall, 393 genes were found to be consistently within the set of the top 500 over-represented genes according to normalized read mapping count in at least 40% of our samples (**Table S2**). Of these 393 genes, ten genes were found to be over-represented by normalized read mapping count across all 12 of the samples considered in the study. The coverage maps of *RPLP0* and *RPL31*, over-represented in all 12 samples, and *SPINT2*, over-represented in 11 samples, demonstrate this coverage bias in these three high expressing transcripts (**Figure 2A,B,C**). We then examined the composition and distribution of reads in some of these highly over-represented transcripts.

### Impact of duplicated reads in amplification-based sequencing

The gene *RPLP0* had much greater total mapping coverage in AS across all twelve samples (**Figure S10**). To aggressively mitigate the effect of amplification in the coverage of this gene, duplicate reads were removed (allowing only 1 read per unique start location) for both technologies as done in previous studies [21], [22]. This resulted in suppression of many of the observed peaks in AS. In contrast, SMS coverage of the gene appeared to be relatively consistent across the length of the *RPLP0* transcript before and after this procedure. This substantial difference in behavior between pre- and post- duplicate read removal for AS in comparison to SMS suggests that amplification is a significant contributory factor in the observed bias. Similar behavior is observed in the *RPL31* and *SPINT2* genes as well.

We considered both alignment locus and read length in our definition of read duplication, allowing one read at each locus with a unique read length. Looking across the transcriptome using this definition of read duplication, we observed a roughly normal distribution along the length of all transcripts captured. A 3-fold difference in the median number of duplicate reads between AS and SMS across all transcripts observed in all samples was maintained across the majority of the transcript length (**Figure 2D**). This pattern of read duplication is similar to that observed in the literature between standard amplification-dependent and amplification-free sequencing methodologies [27]. Removal of duplicate reads, allowing only one read per locus, yielded inconsistent results across the sample set (**Figure S11**). In some cases, the procedure reduced the over-representation in the highest expressing genes, however the bias appeared to remain in other samples. The procedure also drastically reduced the number of usable reads by a median of 47% across the 12 sample set (**Figure S12**). While this naïve methodology of duplicate read removal had some positive effect in reducing the discrepancies between AS and SMS in terms of transcript quantification, the drastic effects it has on the number of usable reads in AS suggests a different approach may be desirable. With this understanding of the impact of duplicated reads, we analyzed the set of recurrently over-represented genes to see if they sequenced biologically interesting categories of genes.

### Gene Ontology analysis of the set of 393 recurrently over-expressed genes

**Figure 3:**
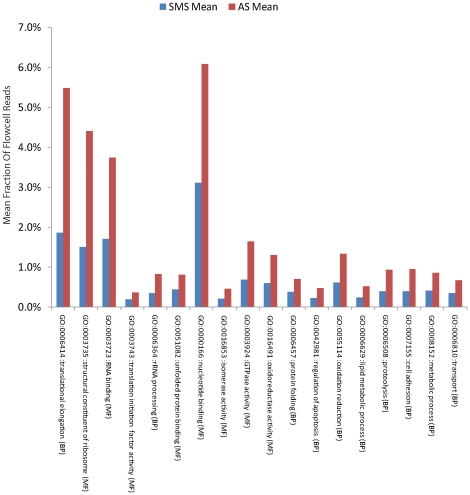
Global representation of Gene Ontology classes in Amplification-based sequencing. GO analysis of the 393 most over-represented genes found using our recurrence analysis in the Molecular Function (MF) and Biological Process (BP) subtrees demonstrates that translational processes and components of the ribosome are over-represented across samples in amplification-based sequencing.

Across the samples, genes associated with the cell's replicative machinery comprised the largest portion of over-represented transcripts by total normalized number of mapping reads in most samples. Gene Ontology analysis of the set of 393 consistently over-represented genes shows that they are components of the cell's translational machinery (**Figure 3**), a class generally found at high levels in all twelve samples used in this evaluation. This again suggests that the amplification procedure implicit in AS library preparation exaggerates a particular bias towards these already-abundant transcripts. The total number of reads falling into each of the classes observed to be over-represented in AS was a mean of 2.23x higher as compared to SMS, although genes overlap between the classes. With less of a focus on high-concentration translational machinery and housekeeping genes, we then attempted to apply SMS in finding gene fusions in the transcriptome.

### Re-discovery of known gene fusions using single-molecule sequencing

We evaluated the applicability of single read SMS in gene fusion discovery by attempting to re-discover known gene fusions in the VCaP cell line, known to harbor *TMPRSS2-ERG*, in a *de novo* process. As shown in **Figure S13**, we first aligned all possible reads against the transcriptome and genome using IndexDP. The non-mapping reads, which harbor chimeras, were subsequently aligned against the transcriptome returning those reads that had a partial alignment of at least 18 nucleotides. The portion of the read that fails to align is defined as the overhang. All reads having the same partial alignments, suggesting a common breakpoint, were clustered. All clusters were then compared to determine if the overhang from one breakpoint region had similarity to the overhang of an independent breakpoint thereby reconstructing the fusion junction. Lastly, all remaining non-mapping reads were aligned against the novel fusion junctions.

**Figure 4:**
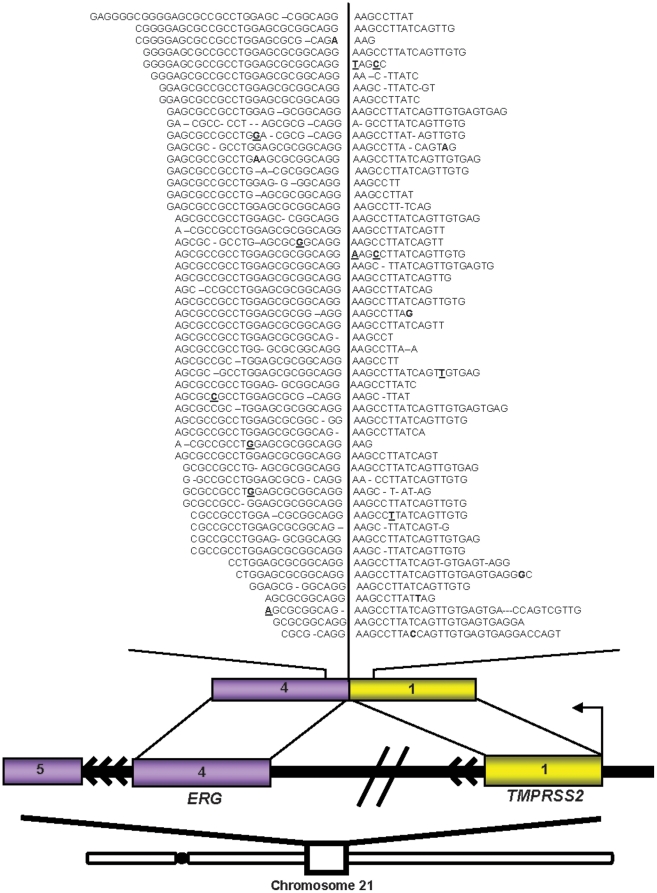
Single molecule sequencing “re-discovers” known gene fusions. Schematic of the intra-chromosomal rearrangement on chromosome 21 fusing *TMPRSS2* (yellow) to *ERG* (purple).

For this purpose, a sample of the VCaP cell line was sequenced more extensively in 2 channels, generating 31,198,128 reads aligned to the transcriptome or genome. The VCaP sample was prepared with one channel each with and without fragmentation. The benchmark fusion between prostate-specific gene *TMPRSS2* and ETS oncogenic family member, *ERG* [28], was found to be covered by 53 reads from generating 65 million reads in the VCaP cell line (**Figure 4**).

## Discussion

This is the first study assessing the performance of RNA-Seq using single-molecule sequencing in comparison to existing amplification-based techniques. While the characteristics of the SMS reads will vary depending on platform, we expect that the distribution of reads across varying transcript concentrations to remain relatively consistent. The SMS technique was able to generate more usable reads in ten of the twelve samples considered in the RNA-Seq quantification and coverage evaluation, producing a mean 78% more reads in these 10 samples. More importantly, these reads tended to be less concentrated at the very highest abundance transcripts as shown in **Figure 1B**, where fraction of total reads mapping to the highest abundance transcripts in SMS are 4% below that of AS. Because the AS technique amasses a large fraction of reads sequencing high-abundance transcripts, detection of lower abundance genes are reduced. The large differences between the highest and second-highest quartile of expressed transcripts suggests that this effect is non-linear as transcript abundance increases in the sample. The wide range of transcript expression in biological samples makes this skewed read distribution of coverage an important factor when profiling mRNAs at the nucleotide level, departing from models that may assume a linear correlation between transcript abundance and sequencing coverage.

The number of duplicated reads observed in the samples across all transcripts was, not surprisingly, 3-fold higher in AS compared to SMS. The removal of duplicate reads is a well-defined procedure in experiments involving DNA sequencing but is less clear-cut when sequencing the transcriptome where varying transcript concentrations naturally lead to reads of identical mRNA segments. This caveat is due to highly expressed transcripts contributing false positive duplicate reads due to random sampling of read start locations along the transcript. However, highly expressed transcripts in SMS would likely generate a large number of these false positives as well. As a result, this source of false positive duplicated reads is unlikely to be the major factor behind the large observed differences in the number of duplicates between AS and SMS. The removal of duplicated reads by filtering out all reads in excess of a single read for a single locus appears to be an incomplete solution that introduces several confounding factors when using single reads. First, the process of removing duplicates is inconsistent, affecting the biased representation of reads in only a subset of the cases we observe. Second, the duplicate removal process also reduced the usable sequence yield from each experimental run by nearly half, although this is an overestimation due to the naïve nature of the method. Finally, these duplicate removal methods impose a peak coverage limit for each transcript that is equivalent to the read length. The naïve process we applied for the elimination of duplicates is most certainly over-aggressive and this issue may be partially alleviated using more sophisticated bioinformatic and statistical methods. However, these processes impose additional confounding factors into the data that SMS avoids entirely due to the direct nature of the sequencing methodology. Alternatively, the use of paired-end reads also produces additional mapping and sequence information that improves the process of duplicate identification and removal. The differences that result from the characteristics of these two methodologies can lead to disparities in the coverage of genes along the spectrum of expression.

Small differences in the distribution of reads at the highest quartile of expressed genes have a large effect on the coverage of the remaining expressed genes. For example, the lowest quartile of all genes seen in both technologies in the VCaP-24 h sample composes 0.4% of the sum total of normalized reads seen in the highest expressed quartile by AS. A 1% reduction in the number of reads used to sequence the highest expressing genes in the forth quartile can be used to triple the coverage of the lowest expressing genes when reads are applied within the set. The result of shifting the read distribution to lower expressing genes is seen between the VCaP-0 h and VCaP AS samples. Both samples yielded a relatively similar number of reads, with 3,636,454 and 3,352,960 reads in VCaP-0 h and VCaP, respectively. However, the VCaP-0 h sample has more than twice the fraction of the total reads falling into the lowest 2 quartiles with 2.2% and 0.9%, in the respective VCaP-0 h and VCaP samples. It comes as no surprise that in the VCaP-0 h sample, we are able to observe 16,813 genes above the 0.3 RPKM noise threshold whereas in VCaP, we only observe 13,866 genes above this threshold. Similarly, the reduced high-abundance coverage bias across variable concentrations allows the SMS approach 2- to 6-fold more coverage in the lower half of all expressed genes. The variable read length of the SMS reads contributes to quantification noise, compared to AS, due to the number of short reads which map ambiguously. These mis-mappings may contribute to the larger number of genes observed at the very lowest expression levels. Examination of the reads mapping to genes only found in SMS shows the presence of more than 30% of long SMS reads (>36 bp in length) in a median of 17% of the genes (approximating the read length distribution across all samples), leaving a 1.7-fold advantage in favor of SMS sensitivity if genes detected with only short 24- to 35-mer reads are all considered detections due to noise. While a significant proportion of this noise is directly attributable to ambiguities in accurately mapping short reads, the presence of long (>36 bp) aligned reads is not a guarantee of transcript presence. In a large number of the cases where detected genes have long reads aligned to them, false positives were attributable to these long reads mapping to repetitive elements or low complexity regions within the transcripts.

Our PCR validation results suggest that using amplification to confirm transcripts exclusively detected by single-molecule sequencing (and missed by AS sequencing) is not ideal, since any sequence that is difficult to amplify will be hard to detect using AS RNA-Seq and hard to validate using an amplification-based system. Therefore, we cannot verify such transcripts unless an amplification-free technology is employed. Sample preparation differences may also contribute to differential representation of transcripts in the sequencing libraries, as AS involves a size selection step that SMS does not. In addition, the two protocols use differing fragmentation procedures which may affect the prevalence of detectable transcript fragments. This is one significant factor that may contribute to the detection of some genes above the noise threshold exclusively by AS. There may be other reasons for differences in the relative representation of transcripts in each technology. Some transcripts may be under-represented because they are hard to capture using SMS. Conversely, the amplification procedure may alter the apparent transcript abundance as some sequences may amplify highly leading to over-representation in AS, which may increase their candidate transcript counts above the noise threshold. For some candidates seen in only one technology, increasing sequencing depth may be the most straightforward solution to the lack of resolution for low abundance transcripts. Some candidates may require modification of the library preparation protocol to ensure sufficient library complexity to capture these low-abundance transcripts. For example, the use of a normalized AS RNA-Seq library preparation protocol or the introduction of a greater amount of input RNA may increase the complexity of the library, possibly enabling higher sensitivity as a result. However, the paucity of published data addressing these topics at this time precludes a thorough examination of potential solutions.

However, while SMS confers the advantages of higher sensitivity and abrogation of issues stemming from read duplication, the technology has a number of confounding characteristics. First, SMS produces reads that are, on average, shorter than their AS counterparts, magnifying the issue of accurately mapping reads to their correct positions. While the inclusion of long 64 bp reads confers an advantage, these are the minority of all reads produced. Approximately 60% of all SMS reads were 36 bp or smaller across all samples. Second, the SMS methodology used in this evaluation produces reads that include randomly introduced gaps due to the incorporation of “dark bases” which do not produce photo-detectable fluorescence. This characteristic requires the use of alignment algorithms that allow for the inclusion of insertions and deletions relative to the reference, and may complicate the detection of structural variation. We also observed a higher proportion of contaminant-alignable reads in SMS compared to AS, although it is unclear whether this is a product of either the sample preparation procedure or a characteristic of the sequencing process.

Altogether, these differences suggest that SMS has advantages in quantitative expression profiling and nucleotide-level assessment such as polymorphism detection in mid- to low- abundance transcripts although the lowest levels of detection are subject to noise due to mapping. However, the log-fold advantage SMS holds may be overcome as rapid advances in sequencing technology result in the production of increasing numbers of usable reads.

## Methods

### Preparation and sequencing of samples

Sequencing libraries for the RNA-Seq evaluation set were prepared from a DU145 cell line (ATCC; HTB-81), an RWPE cell line (ATCC; CRL-11609), an androgen-induced VCaP cell line time course at 0 h, 24 h, 48 h, an identical time course in the LnCaP (ATCC; CRL-1740) cell line, and a tissue sample from a prostate tumor paired with an adjacent normal sample. Sample preparation of the entire 12-sample set included the RNA fragmentation step to ensure consistency. Two replicates of a normal untreated VCaP cell line were run for gene fusion discovery evaluation, one each of fragmented and un-fragmented RNA. The fragmented sample was included in the 12-sample evaluation set. The VCaP cell line was derived from a vertebral metastasis from a patient with hormone-refractory metastatic prostate cancer, and was provided by Ken Pienta (University of Michigan, Ann Arbor, MI). LNCaP or VCaP [29] cells were starved in phenol red free media supplemented with charcoal-dextran filtered FBS and 5% penicillin/streptomycin for 48 h before the addition of 1 nM synthetic androgen (R1881) as indicated. RNA was then isolated using the miRNeasy kit (Qiagen) according to the manufacturer's instructions. Prostate tumor tissue was obtained from the University of Michigan tissue core. Identical samples were submitted for SMS and AS sequencing in all cases with the exception of the VCaP and LnCaP time course samples. The DU145, VCaP, RWPE, as well as the VCaP and LNCaP AS-sequenced time course samples were treated with DNAse. The VCaP and LNCaP time course samples submitted for SMS, as well as the PrCa and PrCa-Adjacent normal samples, were not treated with DNAse during sample preparation. Poly-A containing mRNA for these samples was isolated by two rounds of binding to Sera-Mag Magnetic Oligo(dT) beads, wash and elution in 10 mM Tris buffer pH 7.5, according to manufacturer's instructions (Thermo Scientific, Indianapolis). The purified mRNA was immediately processed for library preparation. The VCaP and LNCaP time course AS sample mRNA was selected with oligodT linked beads according to manufacturer's instructions (Invitrogen).

Amplification-based sequencing was done in paired-end mode run to a minimum of 36 bp per read and trimmed to a minimum of 34 bp to remove low quality bases. For amplification-based sequencing, messenger RNA (2 µg) was fragmented at 85°C for 5 min in a fragmentation buffer (Ambion) and converted to single stranded cDNA using SuperScript II reverse transcriptase (Invitrogen), followed by second-strand cDNA synthesis using *Escherichia coli* DNA polymerase I (Invitrogen). The double stranded cDNA was further processed by Illumina mRNA sequencing Prep kit. Briefly, double-stranded cDNA was end repaired by using T4 DNA polymerase and T4 polynucleotide kinase, monoadenylated using an exo minus Klenow DNA polymerase I (3′to 5′ exonucleotide activity), and ligated with adaptor oligo mix (Illumina) using T4 DNA ligase. The adaptor-ligated cDNA library was then fractioned on a 3% agarose gel, and fragments corresponding to 280–320 bp were excised, purified, and PCR amplified (15 cycles) by Phusion polymerase (NEB). The PCR product was again size selected on a 3% agarose gel by cutting out the fragments in the 300 bp range. The library was then purified with the Qiaquick Minelute PCR Purification Kit (Qiagen) and quantified with the Agilent DNA 1000 kit on the Agilent 2100 Bioanalyzer following the manufacturer's instructions. Library (5–8 pM) was used to prepare flowcells for analysis on the Illumina Genome Analyzer II.

Single-molecule sequencing was done on a Helicos HeliScope in single-read mode, resulting in useful reads ranging between 24 bp and 61 bp for the first set and 25 bp and 64 bp in length in the second set. polyA+ RNA was purified on an RNeasy MinElute column (Qiagen). Then 100 ng of RNA (on average, between 86 ng–130 ng) was heat fragmented by incubation at 95C for 10 minutes or left un-fragmented. First strand cDNA was then made using the SuperScript III reagent kit (Invitrogen, Carlsbad CA) as follows: 500 ng random hexamers, 2 ul of 10 mM dNTP, and DEPC water were added to the RNA up to a volume of 25 ul. The mixture was then incubated at 65C for 5 min and placed directly on ice for 2 minutes. Next, 5 ul 10X buffer, 5 ul 0.1 M DTT, and 10 ul 25 mM MgCl were added to each sample, and the, now 45 ul, sample was incubated at 15C for 30 minutes. After this incubation time 2.5 ul of RNaseOut (100 U), and 2.5 ul of SuperScript III (500 U) were added to each sample and the samples were incubated at 42C for 30 minutes, 55C for 50 minutes, and 85C for 5 minutes. After the reverse transcription reaction, 1 ul RNase H and 1 ul of RNase I were added to each sample, followed by a 30 minute incubation at 37C.

Samples were twice purified on DyeEx columns (Qiagen). cDNA samples were then Poly-A tailed using the Helicos DGE assay reagent kit (Helicos, Cambridge MA), and the terminal transferase kit (NEB, Ipswich MA) as follows: 5 ul Helicos Tailing control Oligonucleotide A was added to 20 ul of each cDNA and the volume was adjusted to 35.5 ul with water. This mixture was then denatured for 5 minutes at 95C and placed directly on ice for 2 minutes. Then, 5 ul 2.5 mM CoCl, 5 ul 10X terminal transferase buffer, 2 ul Helicos polyA tailing dATP, and 1.2 ul terminal transferase (24 U) were added to each samples, followed by incubation at 42C for 1 hour, and then 70C for 10 minutes. After the tailing reaction the samples were 3′ blocked as follows: samples were denatured for 5 minutes at 95C and placed directly on ice for 2 minutes, 300 pmoles biotin-dideoxy ATP (Perkin Elmer, Waltham MA) and 1.2 ul terminal transferase (24 U) were then added, followed by 1 hour incubation at 37C, and a final 10 minute heat inactivation step at 70C. 3′ biotinylation of samples was used to assess sample molarity to inform HeliScope sample-loading for the sequencing reaction (according to manufacturer's instructions).

### Alignment of reads

The first read of AS read pairs was used in this study to compare to the single reads derived from SMS. SMS reads were aligned with the IndexDP aligner, while amplification-based sequencing reads were aligned with both the Bowtie and IndexDP aligners as shown in **Figure S2**. IndexDP alignments were filtered by NScore, defined as (5*#_match-4*#_error)/read_length) with a minimum of score 4, reporting at most 25 alignments per read. Reads between 24 bp and 57 bp and 25 bp and 64 bp in length were used for sets 1 and 2, respectively. Bowtie was set to report alignments with at most two mismatches within a 32-base seed region, reporting at most 25 multiple alignments per read. The first base of all AS reads was trimmed to maximize quality. Single-best quality alignments were derived using Bowtie by setting the –best and –k 1 parameters to report only the single highest quality alignment per read. Reads were aligned to the set of UCSC transcripts defined in hg18, downloaded from the UCSC Genome Browser at http://genome.ucsc.edu. Known contaminants were also included in the set of references. Bowtie alignments included references for mitochondrial DNA, adapter sequence, and ribosomal RNA. IndexDP alignments included references for poly-A, poly-T, poly-C, and poly-G oligomers. Re-alignment of AS reads using IndexDP was done using the same parameters as SMS reads, using the full length of the read. Reads from the PrCa sample were trimmed to 50 bp from 75 bp to meet technical limitations of the alignment program. Sequence reads from this study have been deposited into the NCBI Short Read Archive with accession number SRA028835.1.

### Duplicate read removal

Duplicate reads were removed from the data by analyzing the alignments to each UCSC transcript in the transcriptome reference. One read was allowed to align at each start locus (with and without consideration of read length). Reads with alignments to locations along the reference transcript in excess to those were marked as duplicates and removed from the data set.

### Relative quantification of genes and coverage calculation

Reads aligning to each UCSC transcript were counted at transcript level resolution and then summarized at the gene level using transcript to gene symbol mappings from the kgXref table downloaded from the UCSC Genome Browser at http://genome.ucsc.edu. Reads aligning to the known contaminant references were marked and not considered in the analysis. Genes were quantified using only the single-best mapping methodology. Single-best mappings were derived from IndexDP alignments by choosing alignments with the highest NScore, or an alignment randomly picked from the set of highest scores when multiple alignments are present with the same NScore value. Gene-level RPM values were derived by summing the number of aligned reads from each gene's constituent transcript isoforms and dividing by the total number of usable reads. Read sums were calculated using R Statistical Environment [30]. RPKM values were computed for each observed UCSC transcript and summed for all isoforms of a gene to derive a gene-level RPKM expression value. Coverage levels were calculated by summing the read lengths of all reads aligning to all isoforms of each gene and dividing by the mean isoform length.

### Detection of genes observed in a single technology

We derived a list of genes observed in only SMS or AS for the DU145 samples in this study by comparing the mean gene-level RPKM expression values of each pair of samples run on AS and SMS. A list of candidates was nominated by then sorting the list of genes with expression values above the noise threshold in SMS and below the threshold in AS by the observed differences. These genes were evaluated for mis-mappings by examining secondary and alternate alignments of the reads aligning to each candidate as shown in **Figure S14**. The list was filtered to remove genes detected only by short reads and the top 50 remaining genes manually evaluated to have well-defined HUGO names, diffuse read distribution along the transcript length, and the presence of long (>36 bp) reads in both SMS technical replicates.

### Validation of Detected Single-Technology Transcripts by PCR

RNA was extracted from the cells using Qiazol based on Qiagen's miRNeasy Minikit following the manufacturer's instructions (Qiagen). 1 µg of total RNA was reverse transcribed into cDNA using SuperScript III (Invitrogen) in the presence of oligo dT and random primers. Quantitative PCR was carried out by Taqman assay method using gene specific primers and probes from the Universal Probe Library (UPL), Human (Roche) as the internal oligonucleotide, according to manufacturer's instructions. GAPDH was used as housekeeping control gene for UPL based Taqman assay (Roche), as per manufacturer's instructions.

All assays were performed in duplicate using the primer sequences in **Table S5**.

### Gene Ontology analysis of reads

Gene Ontology (GO) analysis of over-represented genes was done in order to assess the most highly represented GO classes and determine the relative abundance of reads attributable to each GO class. This analysis was done with GeneCoDis2 tool [31]. Single GO classes resulting from this process were evaluated for their representation in terms of fraction of total sequenced reads across the 12-sample set. Relative representation of reads attributable to each GO class was done by summing the number of single-best mapping alignments for each gene in each GO class as defined in the GO annotations for *Homo Sapiens*, downloaded from http://www.geneontology.org and dividing the total by the total number of reads in each sample.

### Gene fusion discovery in single-molecule sequencing

The VCaP cell line was sequenced in two additional channels to evaluate the suitability of single molecule sequencing for the task of gene fusion detection. This was done by mining the reads in an effort to re-discover known gene fusions. All possible reads were first aligned against the transcriptome and genome using IndexDP. Non-mapping reads, which harbor chimeras, were subsequently aligned against the transcriptome returning those reads that had a partial alignment of at least 18 nucleotides. All reads having the same partial alignments, suggesting a common breakpoint, were clustered. All clusters were then compared to see determine if the overhang (portion of the read that fails to align) from one breakpoint region had similarity to the overhang of an independent breakpoint, thereby reconstructing the fusion junction. Finally, all remaining non-mapping reads were aligned against the novel fusion junctions. This de novo approach enabled the re-discovery of the TMPRSS2-ERG gene fusion across two channels of SMS reads.

## Supporting Information

Figure S1 **Single-molecule mRNA-sequencing.** mRNAs are purified using poly-A selection and then fragmented. 1st-strand cDNA is synthesized from the fragmented mRNA, and then poly-A tailed using terminal transferase. Polyadenylated cDNA fragments are hybridized to poly-T oligomers bound to a glass substrate, excess A bases are “filled,“ and then “locked” with an A, C, or G base attached to a virtual terminator. The sequencing process then occurs with repeated cycles of virtual terminator cleavage, bases addition, and image readout.
(TIF)

Figure S2 **Read alignment with Bowtie and IndexDP.** Bowtie was used for amplification-based sequencing read alignment and IndexDP for single molecule read alignment. While different in their parameters, the effective alignments and specificity between the aligners are similar, although Bowtie has a slightly higher cutoff.
(TIF)

Figure S3 **Length distribution of aligned SMS reads.** Aligned SMS read lengths varied between 24 bp to 57 bp in our first set of samples and 25 bp to 63 bp in our second set. The majority of reads are between 25 bp and 45 bp in length.
(TIF)

Figure S4 **Sample Profiling Reproducibility in SMS and AS.** Bowtie was used for amplification-based sequencing read alignment and IndexDP for single molecule read alignment. Pearson correlation for log2-transformed, normalized tag counts is r = 0.98 for both SMS and AS.
(TIF)

Figure S5 **Log2 correlation between amplification-based and single-molecule sequencing.** Log2 correlation between single-molecule and amplification-based RNA-Seq single-best read mappings in these samples show that in broad terms the two sequencing methods yield similar results, suggesting the observed bias is not due to sample differences.
(TIF)

Figure S6 **Correlation between IndexDP and Bowtie alignment of amplification-based sequencing reads.** The correlation between Bowtie and IndexDP within the subset of samples was relatively high, with Pearson correlation values above r = 0.95 in all samples.
(TIF)

Figure S7 **IndexDP realignment of amplification-based sequencing reads.** Alignment of amplification-based sequencing reads using the IndexDP alignment tool used to align single-molecule reads shows persistence of the observed bias in amplification-based technology. This provides evidence that the alignment method is not responsible for this bias towards high-concentration transcripts.
(TIF)

Figure S8 **Unique gene detection in AS and SMS across threshold values, by transcript length.** The pattern of increased sensitivity in SMS is uniform as the baseline noise level is varied from 0.1 to 3.0 RPKM. Low representation by short transcripts show that this effect is not due to the lack of a size-selection step in SMS.
(TIF)

Figure S9 **Expression values of validation candidate genes showing amplification.** Out of the set of genes chosen for RT-PCR validation for their detection over the 0.3 RPKM noise threshold by only SMS, diffuse read alignment pattern, and the presence of long reads aligned to their transcripts, these ten genes showed detectable amplification.
(TIF)

Figure S10 **RPLP0 coverage in other samples.** Coverage plots of the over-represented gene RPLP0 in the LNCaP-24 h, LNCaP-48 h, VCaP-24 h, VCaP-48 h, and PrCa-Met samples show that this gene is often more highly sequenced using the amplification-based method.
(TIF)

Figure S11 **Quantile-quantile plot of AS and SMS reads with duplicates removed.** Reads in excess of a single read per aligned locus were removed from both AS and SMS data sets. The result of this procedure was inconsistent across the data set; some samples saw reduced representation of high expressing genes while the high-concentration bias remained in others.
(TIF)

Figure S12 **Effect of duplicate removal in AS.** Reads in excess of a single read per aligned locus were removed from both AS and SMS data sets, resulting in (A) a median 47% drop in the number of usable reads across the 12 samples in the evaluation set and (B) the loss of dynamic range for genes in with high coverage levels.
(TIF)

Figure S13 **Gene Fusion Discovery Using SMS Reads.** All possible reads were aligned against the transcriptome and genome using IndexDP. The set of non-mapping reads (some of which harbor chimeras) were subsequently aligned against the transcriptome, returning reads that had a partial alignment of at least 18 nucleotides. All reads having the same partial alignments, suggesting a common breakpoint, were clustered. All clusters were then compared to determine if the non-aligning “overhang” portion of the read from one breakpoint region had similarity to the overhang of an independent breakpoint, thereby reconstructing the fusion junction. Finally, all remaining non-mapping reads were aligned against the candidate novel fusion junctions.
(TIF)

Figure S14 **Alternate mappings for genes detected by SMS only in DU145.** We analyzed alternate mappings for the reads attributable to each of the nine genes we observed to be detectable only by SMS in DU145 using reads from both replicates. In all nine cases, reads mapped most strongly to the genes of interest, suggesting that the detection of these genes is not an artifact of mismapping. The top 20 alternate mappings, ordered by mapping read count, are shown in the graph.
(TIF)

Table S1 Sample statistics in (A) amplification-based sequencing and (B) single-molecule sequencing technologies.
(TIF)

Table S2 Recurrently over-represented genes in amplification-based sequencing in ten or more samples. Of the 393 genes are recurrently within the top 500 over-represented genes by total read count in five (40%) or more samples, these 59 are seen most often, occurring in at least 10 samples.
(TIF)

Table S3 Sum of normalized expression values per quartile by sample in AS and SMS. We observe that the number of reads aligning to transcripts seen in the third and fourth quartiles is consistently greater in SMS than AS across the sample set.
(TIF)

Table S4 Gene-level read coverage of observed transcripts. (A) and (B) illustrate the number of genes with coverage values at various depths in single molecule and amplification-based sequencing, respectively.
(TIF)

Table S5 Primers used for validating transcripts seen only by SMS. All experiments were performed in duplicate using two primer pairs per candidate gene when possible.
(TIF)
