# Supplementary figures and images for: A Comparison of Single Molecule and Amplification Based Sequencing of Cancer Transcriptomes
Source: PLoS One. 2011 Mar 1;6(3):e17305. doi: 10.1371/journal.pone.0017305 (PMC3046973; doi:10.1371/journal.pone.0017305)

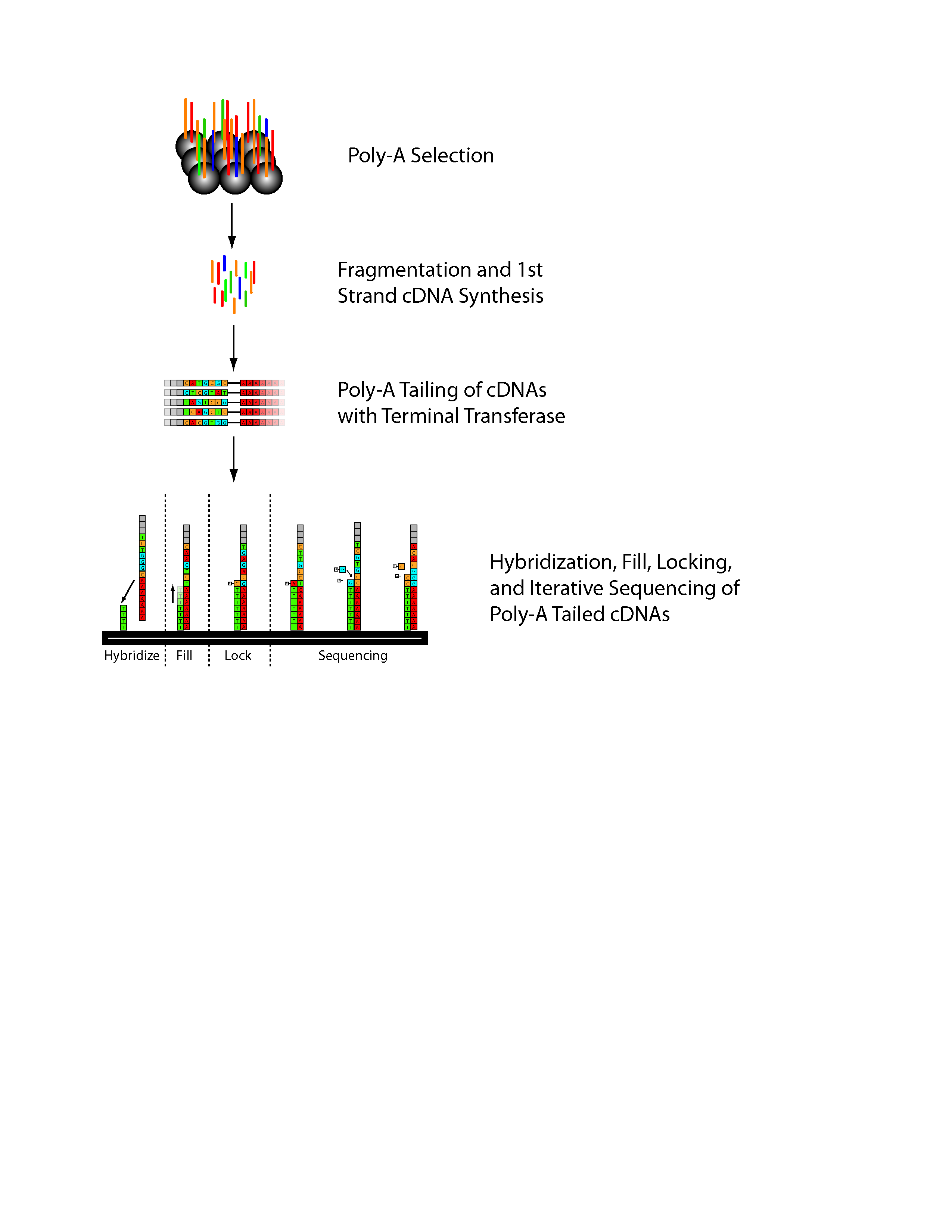

Supplement: Figure S1 — Single-molecule mRNA-sequencing. mRNAs are purified using poly-A selection and then fragmented. 1st-strand cDNA is synthesized from the fragmented mRNA, and then poly-A tailed using terminal transferase. Polyadenylated cDNA fragments are hybridized to poly-T oligomers bound to a glass substrate, excess A bases are “filled,“ and then “locked” with an A, C, or G base attached to a virtual terminator. The sequencing process then occurs with repeated cycles of virtual terminator cleavage, bases addition, and image readout. (TIF) [file pone.0017305.s001.tif]

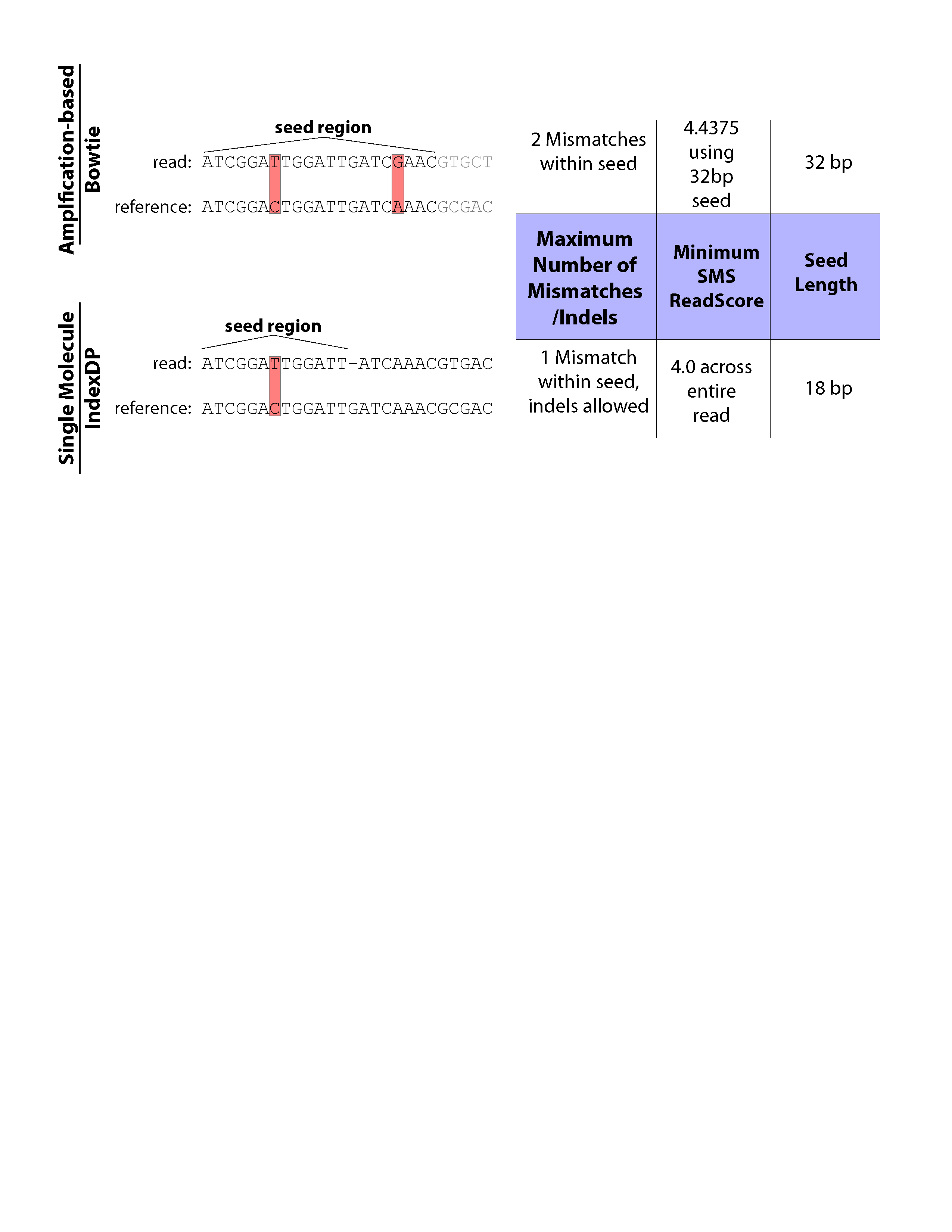

Supplement: Figure S2 — Read alignment with Bowtie and IndexDP. Bowtie was used for amplification-based sequencing read alignment and IndexDP for single molecule read alignment. While different in their parameters, the effective alignments and specificity between the aligners are similar, although Bowtie has a slightly higher cutoff. (TIF) [file pone.0017305.s002.tif]

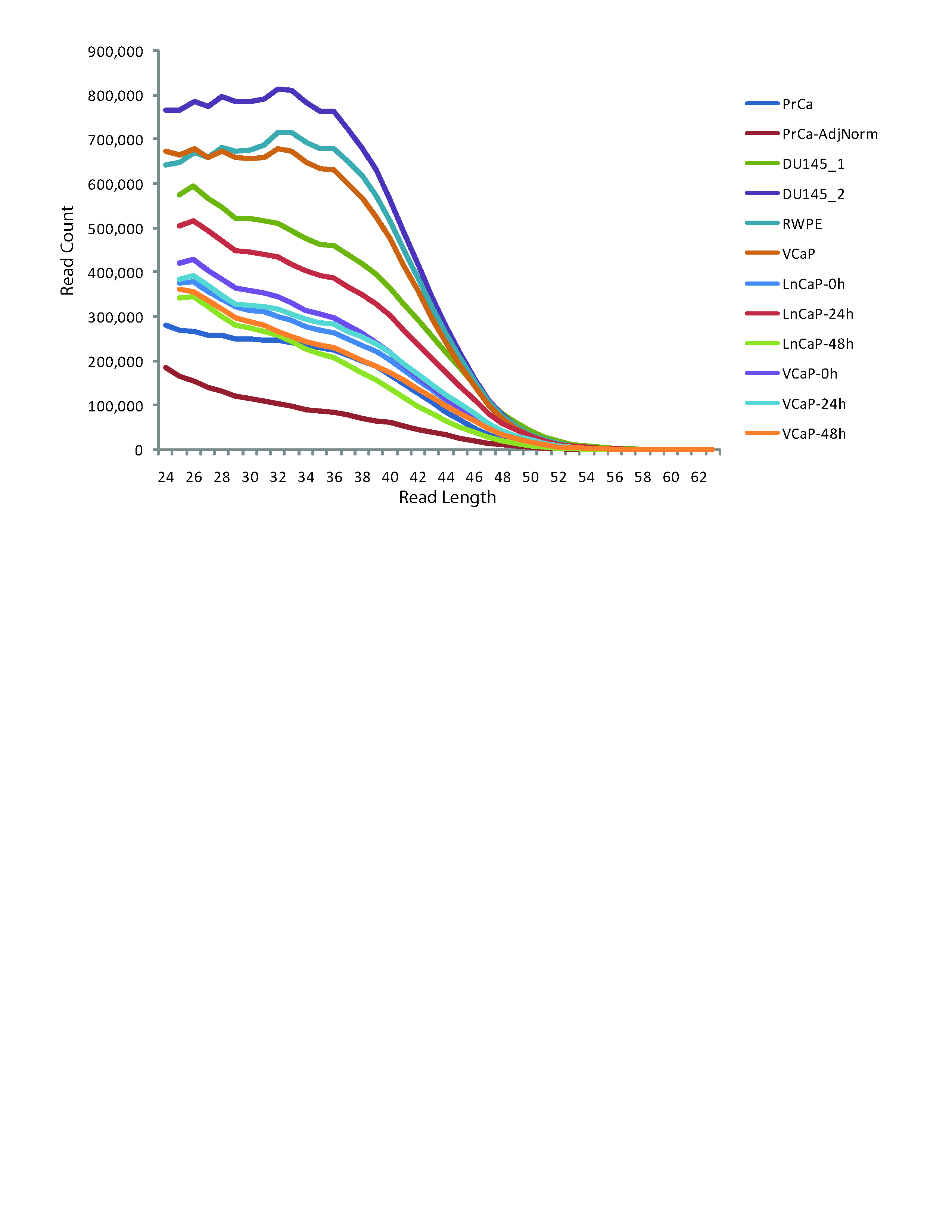

Supplement: Figure S3 — Length distribution of aligned SMS reads. Aligned SMS read lengths varied between 24 bp to 57 bp in our first set of samples and 25 bp to 63 bp in our second set. The majority of reads are between 25 bp and 45 bp in length. (TIF) [file pone.0017305.s003.tif]

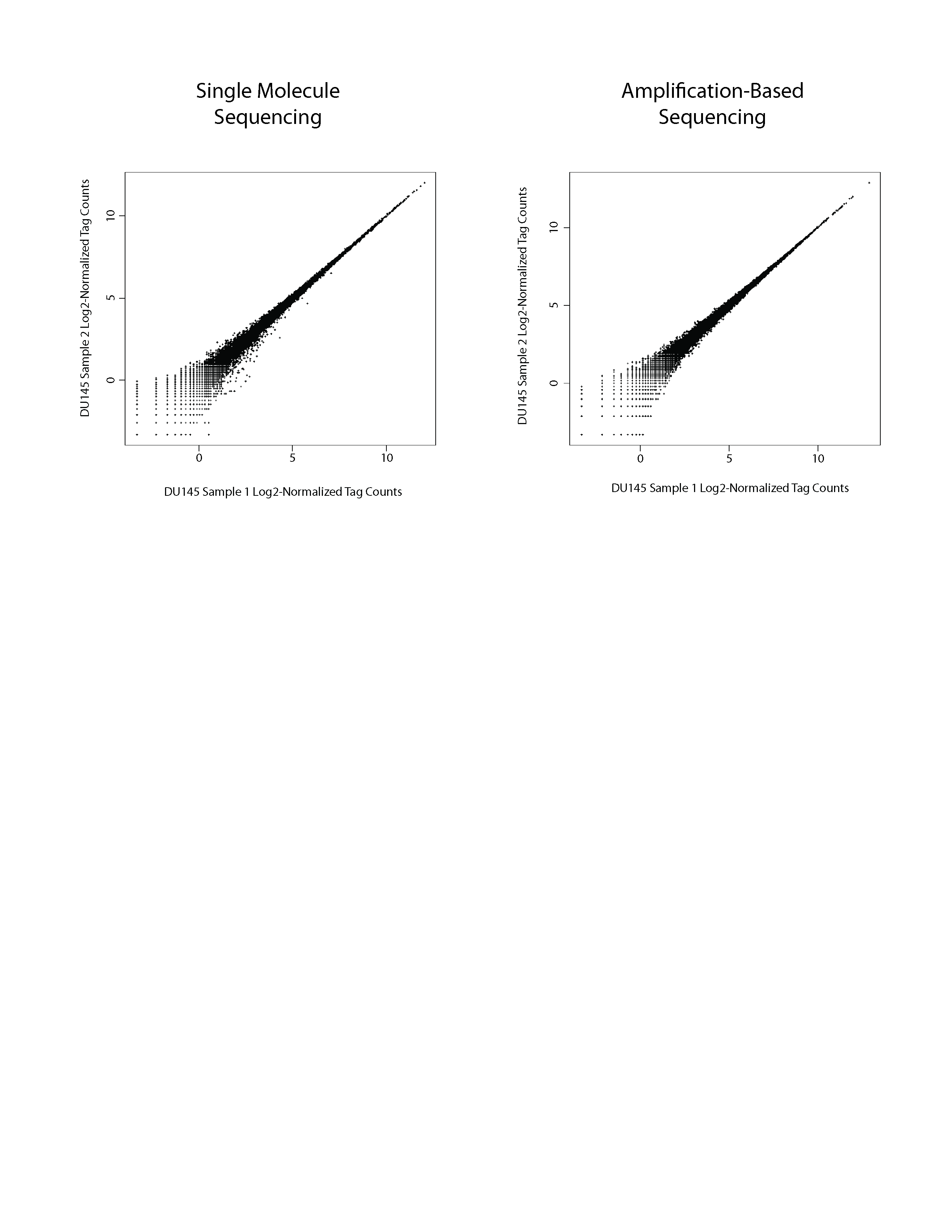

Supplement: Figure S4 — Sample Profiling Reproducibility in SMS and AS. Bowtie was used for amplification-based sequencing read alignment and IndexDP for single molecule read alignment. Pearson correlation for log2-transformed, normalized tag counts is r = 0.98 for both SMS and AS. (TIF) [file pone.0017305.s004.tif]

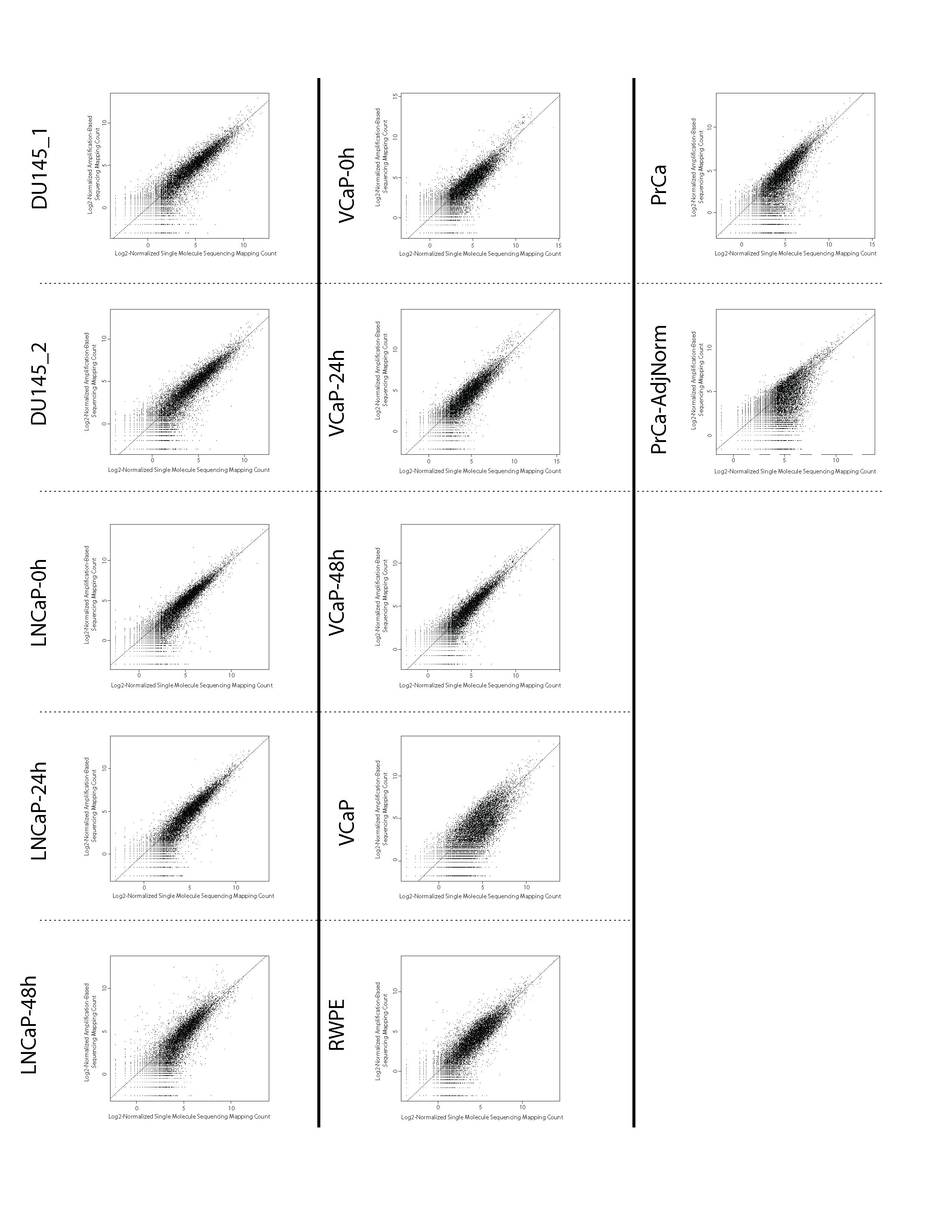

Supplement: Figure S5 — Log2 correlation between amplification-based and single-molecule sequencing. Log2 correlation between single-molecule and amplification-based RNA-Seq single-best read mappings in these samples show that in broad terms the two sequencing methods yield similar results, suggesting the observed bias is not due to sample differences. (TIF) [file pone.0017305.s005.tif]

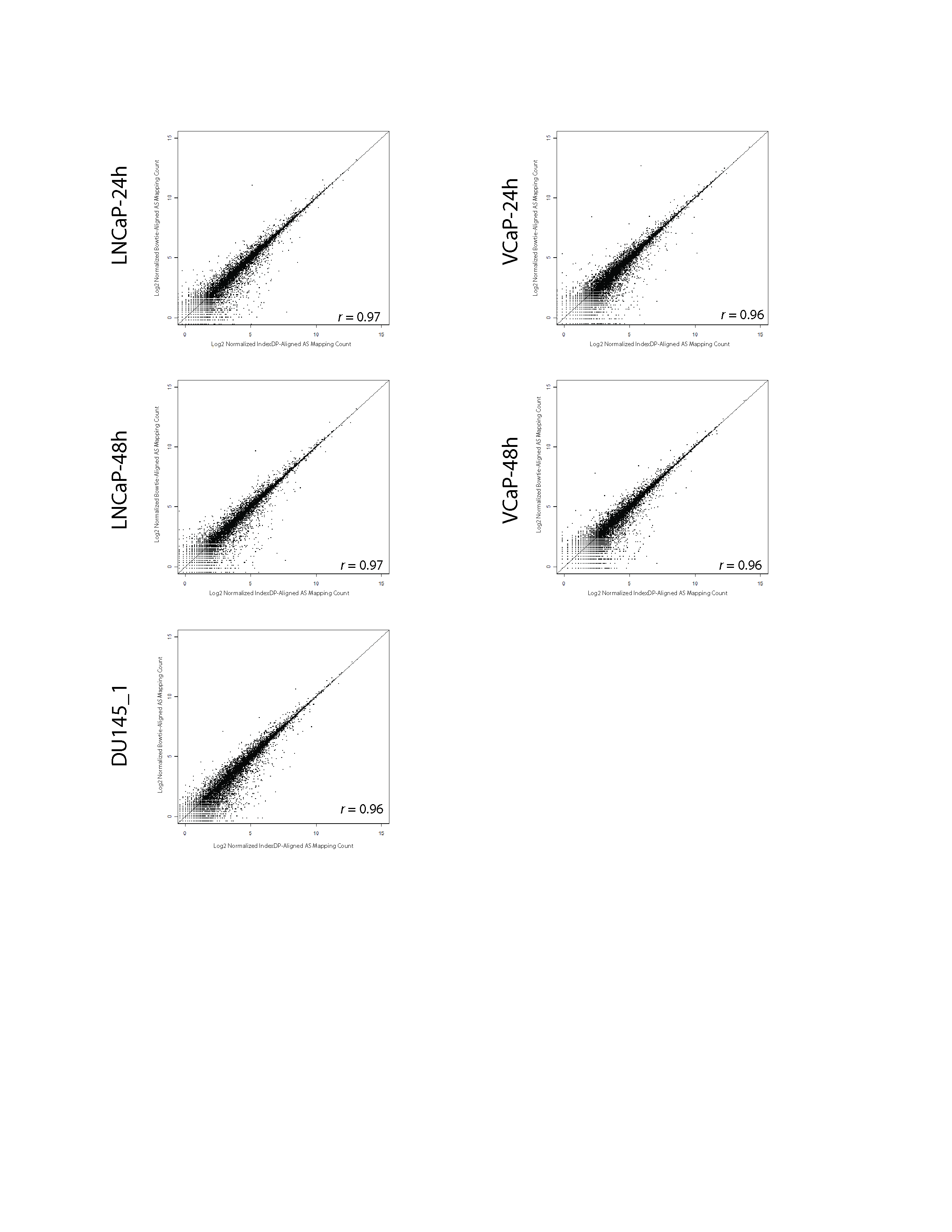

Supplement: Figure S6 — Correlation between IndexDP and Bowtie alignment of amplification-based sequencing reads. The correlation between Bowtie and IndexDP within the subset of samples was relatively high, with Pearson correlation values above r = 0.95 in all samples. (TIF) [file pone.0017305.s006.tif]

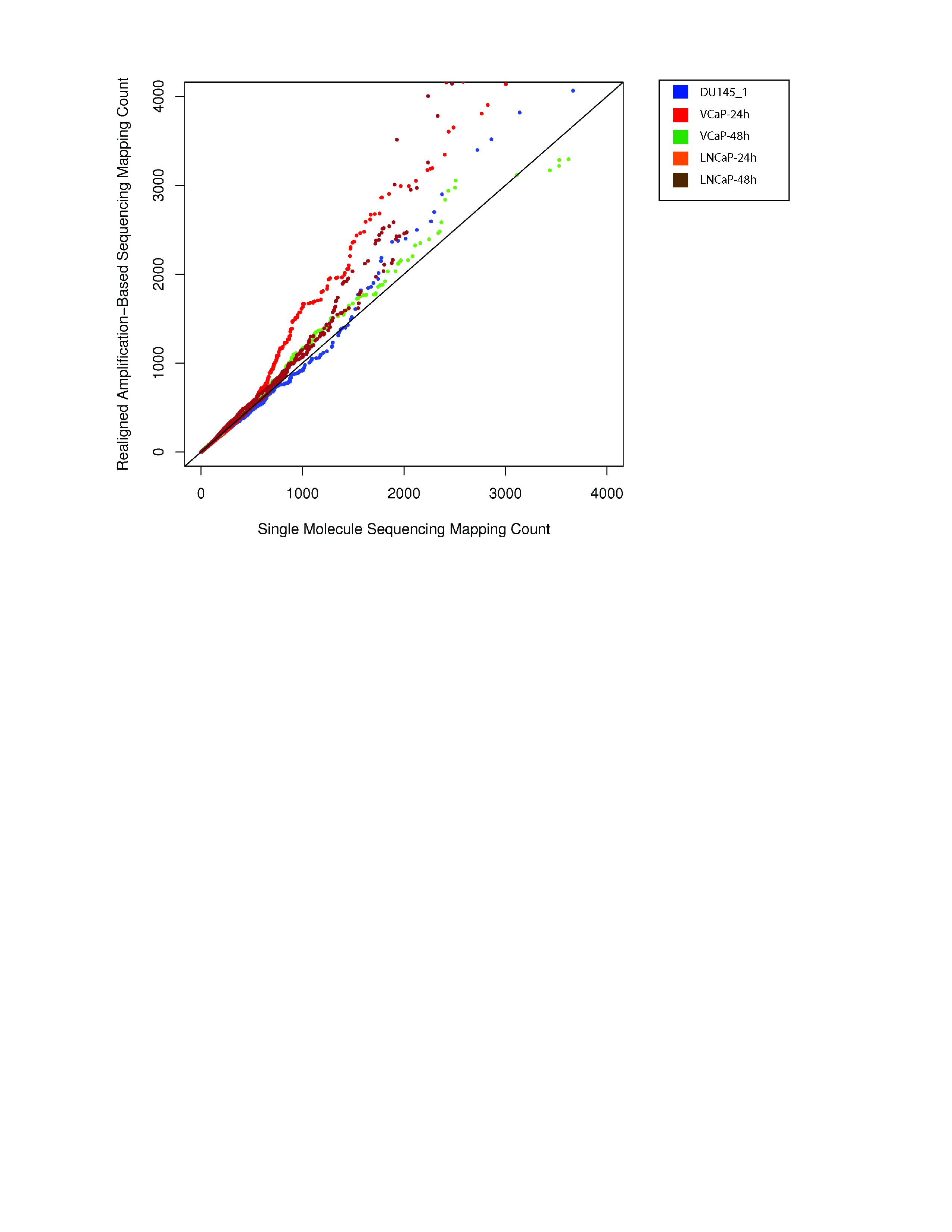

Supplement: Figure S7 — IndexDP realignment of amplification-based sequencing reads. Alignment of amplification-based sequencing reads using the IndexDP alignment tool used to align single-molecule reads shows persistence of the observed bias in amplification-based technology. This provides evidence that the alignment method is not responsible for this bias towards high-concentration transcripts. (TIF) [file pone.0017305.s007.tif]

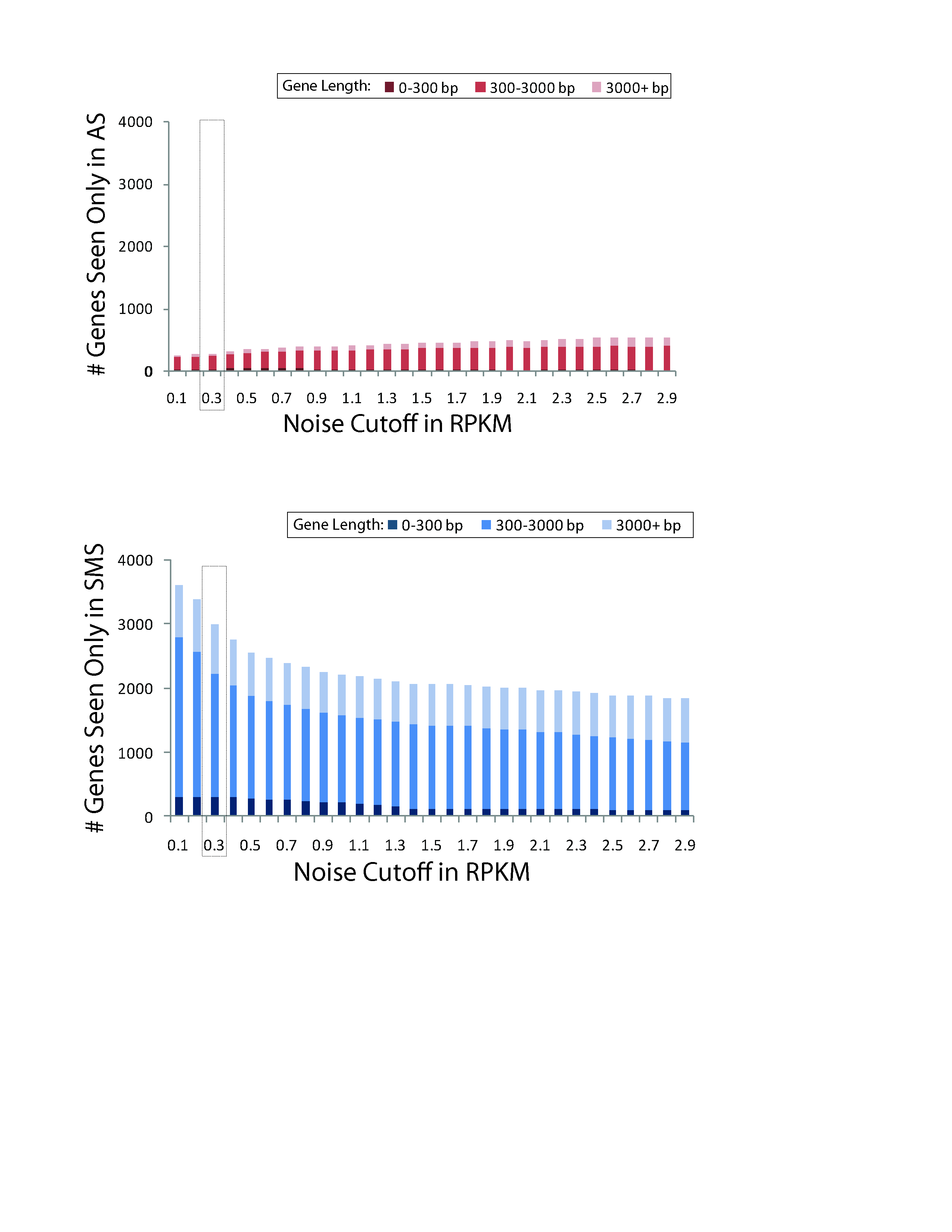

Supplement: Figure S8 — Unique gene detection in AS and SMS across threshold values, by transcript length. The pattern of increased sensitivity in SMS is uniform as the baseline noise level is varied from 0.1 to 3.0 RPKM. Low representation by short transcripts show that this effect is not due to the lack of a size-selection step in SMS. (TIF) [file pone.0017305.s008.tif]

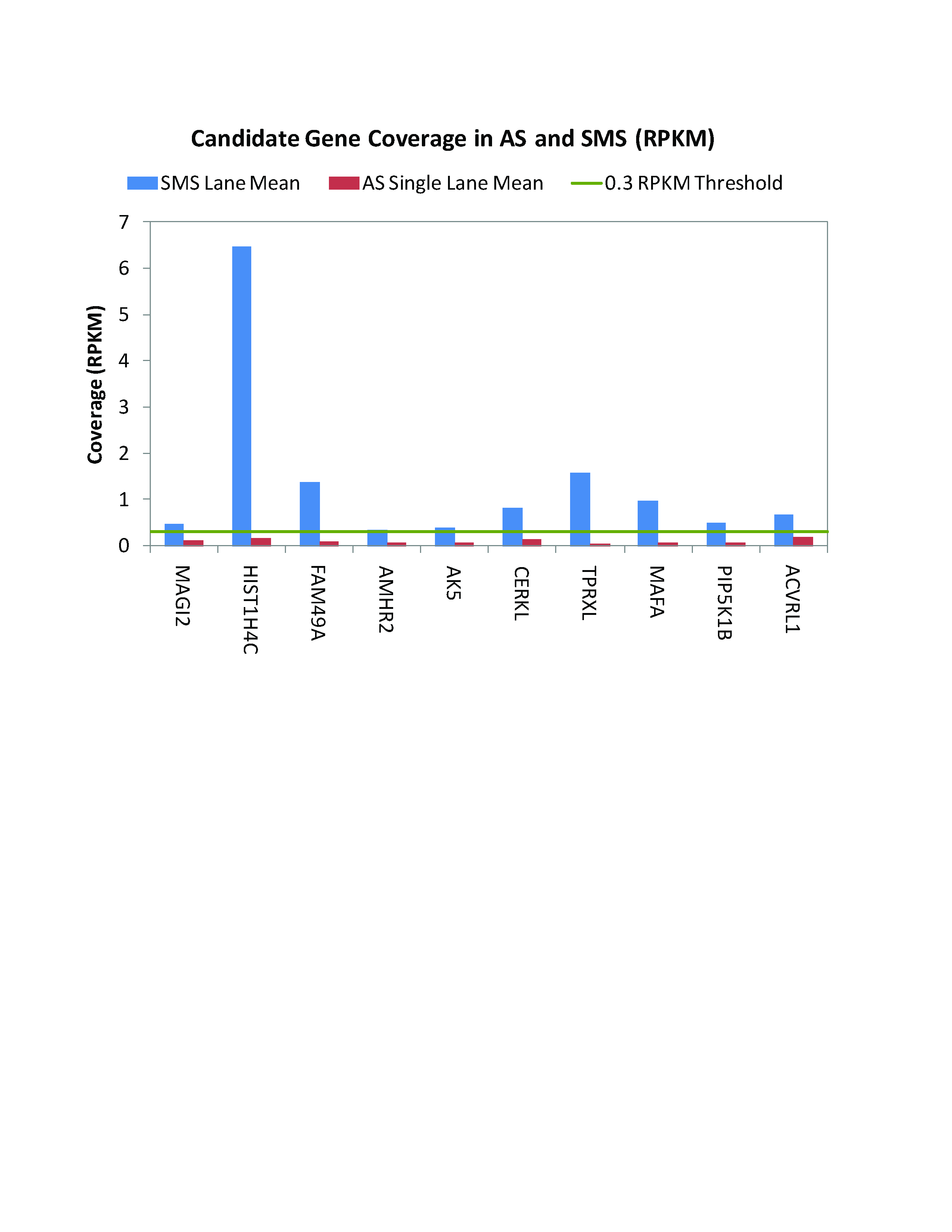

Supplement: Figure S9 — Expression values of validation candidate genes showing amplification. Out of the set of genes chosen for RT-PCR validation for their detection over the 0.3 RPKM noise threshold by only SMS, diffuse read alignment pattern, and the presence of long reads aligned to their transcripts, these ten genes showed detectable amplification. (TIF) [file pone.0017305.s009.tif]

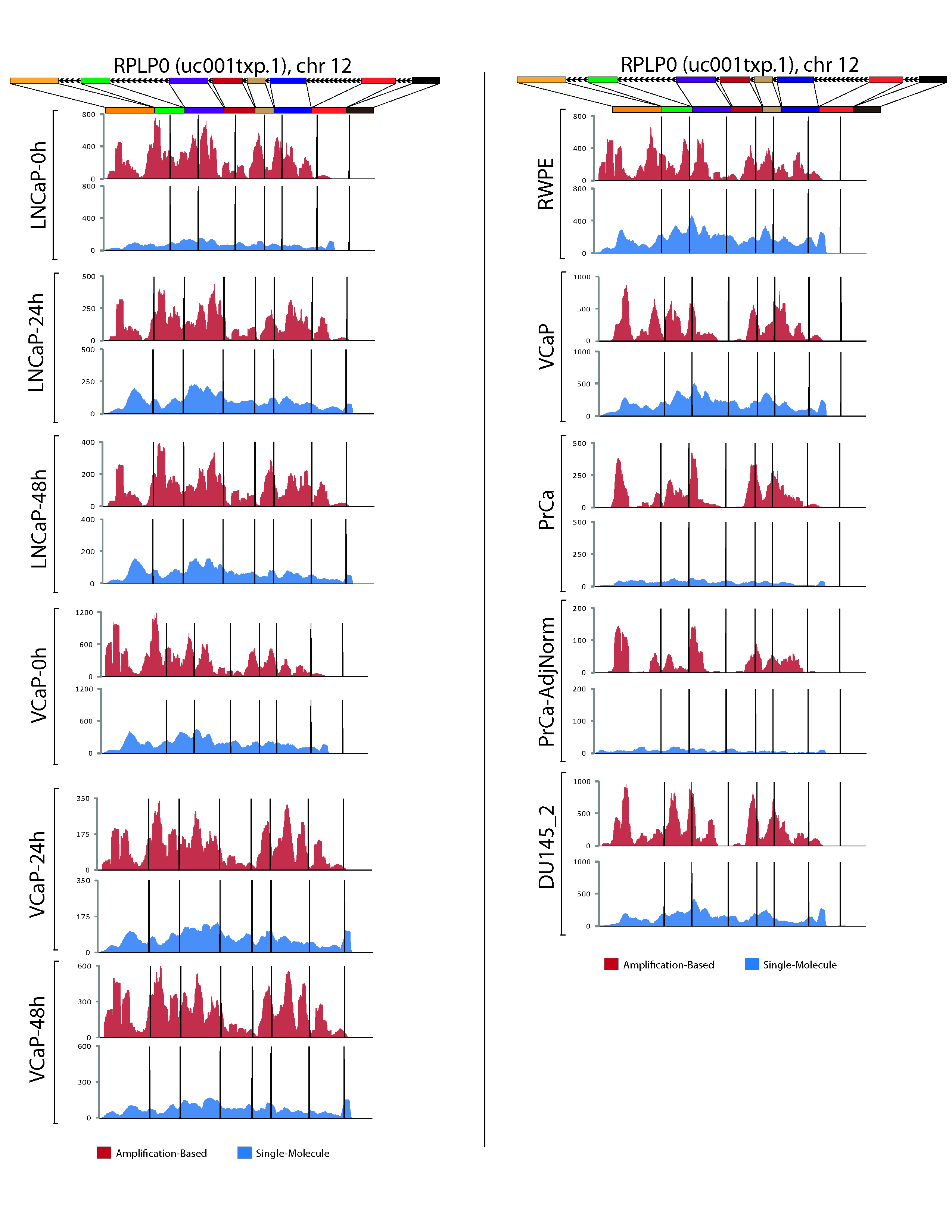

Supplement: Figure S10 — RPLP0 coverage in other samples. Coverage plots of the over-represented gene RPLP0 in the LNCaP-24 h, LNCaP-48 h, VCaP-24 h, VCaP-48 h, and PrCa-Met samples show that this gene is often more highly sequenced using the amplification-based method. (TIF) [file pone.0017305.s010.tif]

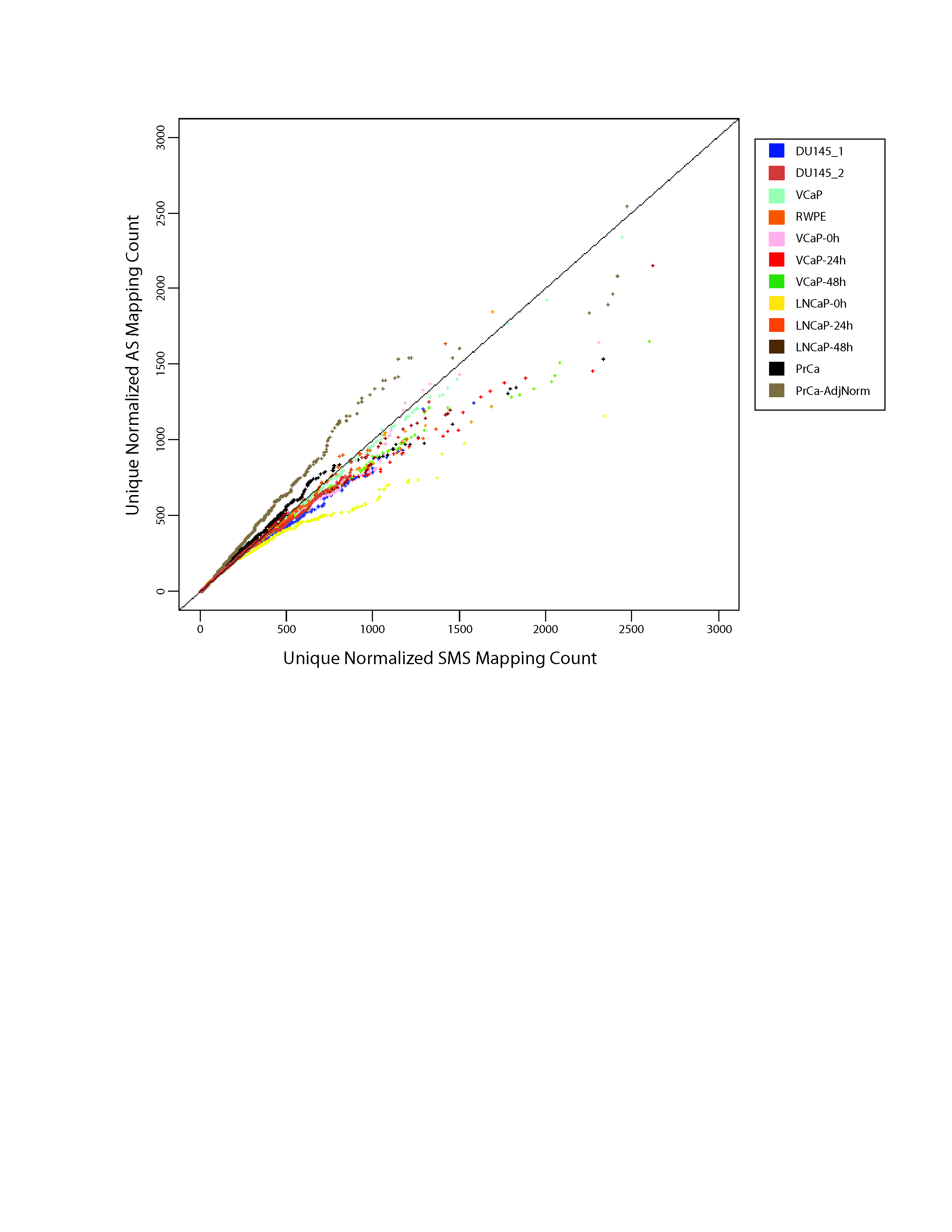

Supplement: Figure S11 — Quantile-quantile plot of AS and SMS reads with duplicates removed. Reads in excess of a single read per aligned locus were removed from both AS and SMS data sets. The result of this procedure was inconsistent across the data set; some samples saw reduced representation of high expressing genes while the high-concentration bias remained in others. (TIF) [file pone.0017305.s011.tif]

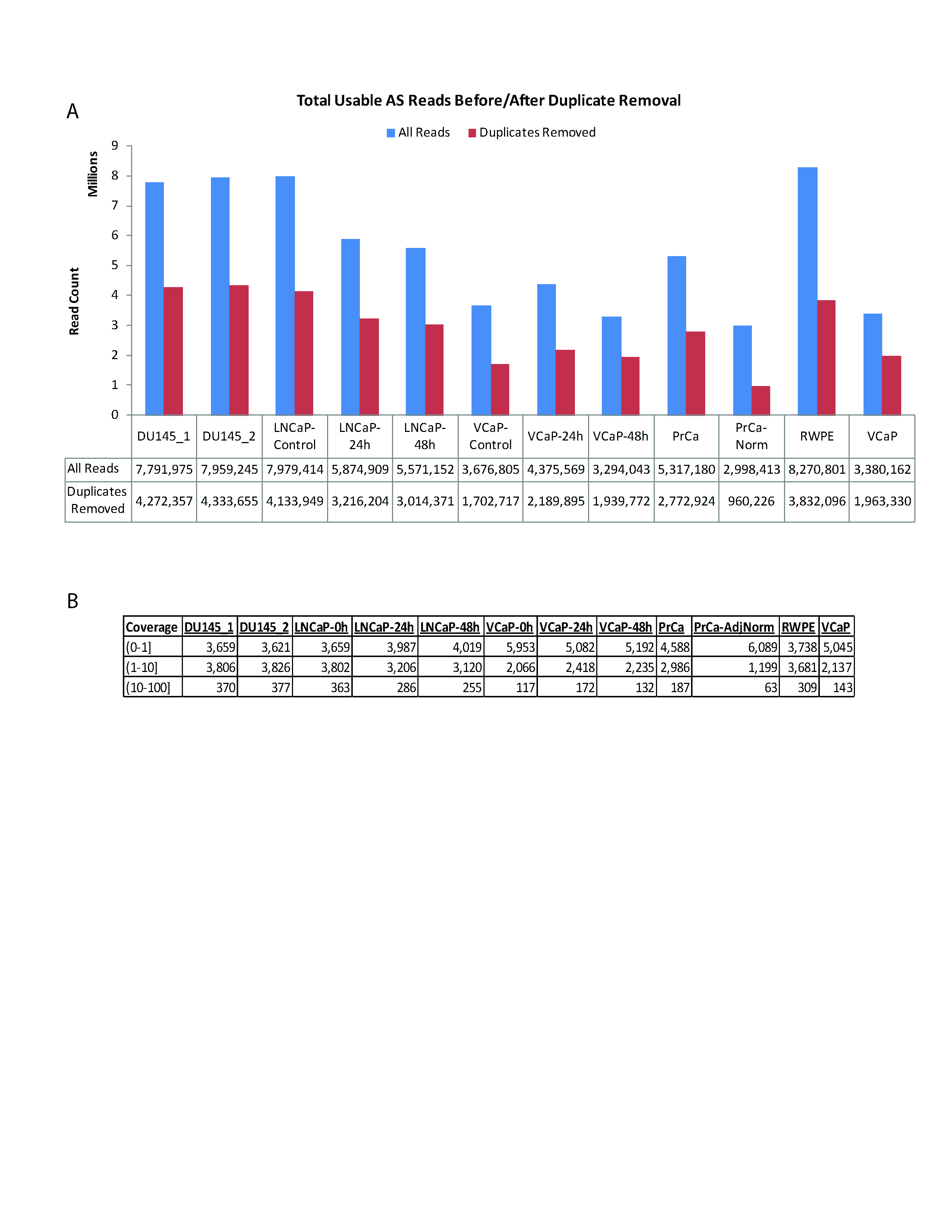

Supplement: Figure S12 — Effect of duplicate removal in AS. Reads in excess of a single read per aligned locus were removed from both AS and SMS data sets, resulting in (A) a median 47% drop in the number of usable reads across the 12 samples in the evaluation set and (B) the loss of dynamic range for genes in with high coverage levels. (TIF) [file pone.0017305.s012.tif]

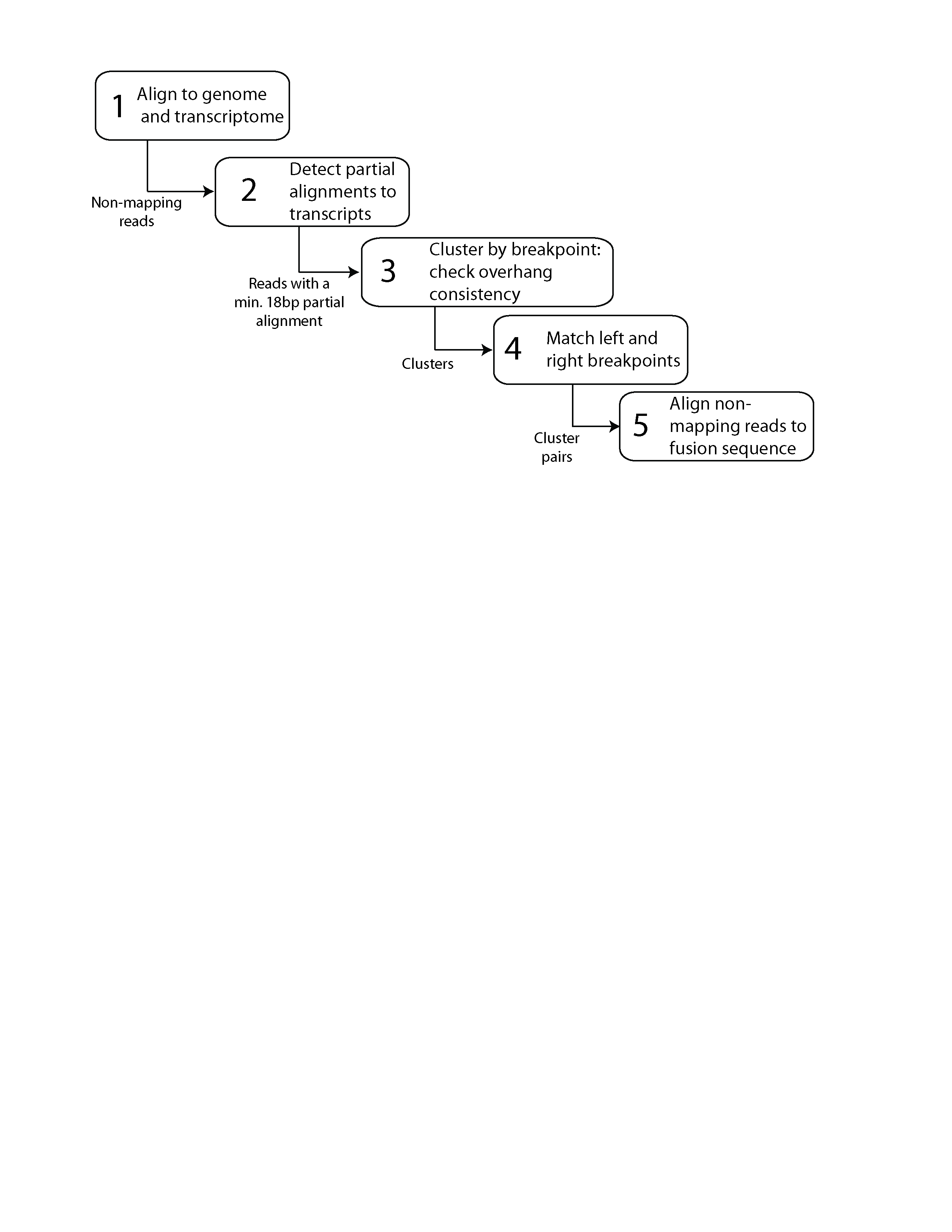

Supplement: Figure S13 — Gene Fusion Discovery Using SMS Reads. All possible reads were aligned against the transcriptome and genome using IndexDP. The set of non-mapping reads (some of which harbor chimeras) were subsequently aligned against the transcriptome, returning reads that had a partial alignment of at least 18 nucleotides. All reads having the same partial alignments, suggesting a common breakpoint, were clustered. All clusters were then compared to determine if the non-aligning “overhang” portion of the read from one breakpoint region had similarity to the overhang of an independent breakpoint, thereby reconstructing the fusion junction. Finally, all remaining non-mapping reads were aligned against the candidate novel fusion junctions. (TIF) [file pone.0017305.s013.tif]

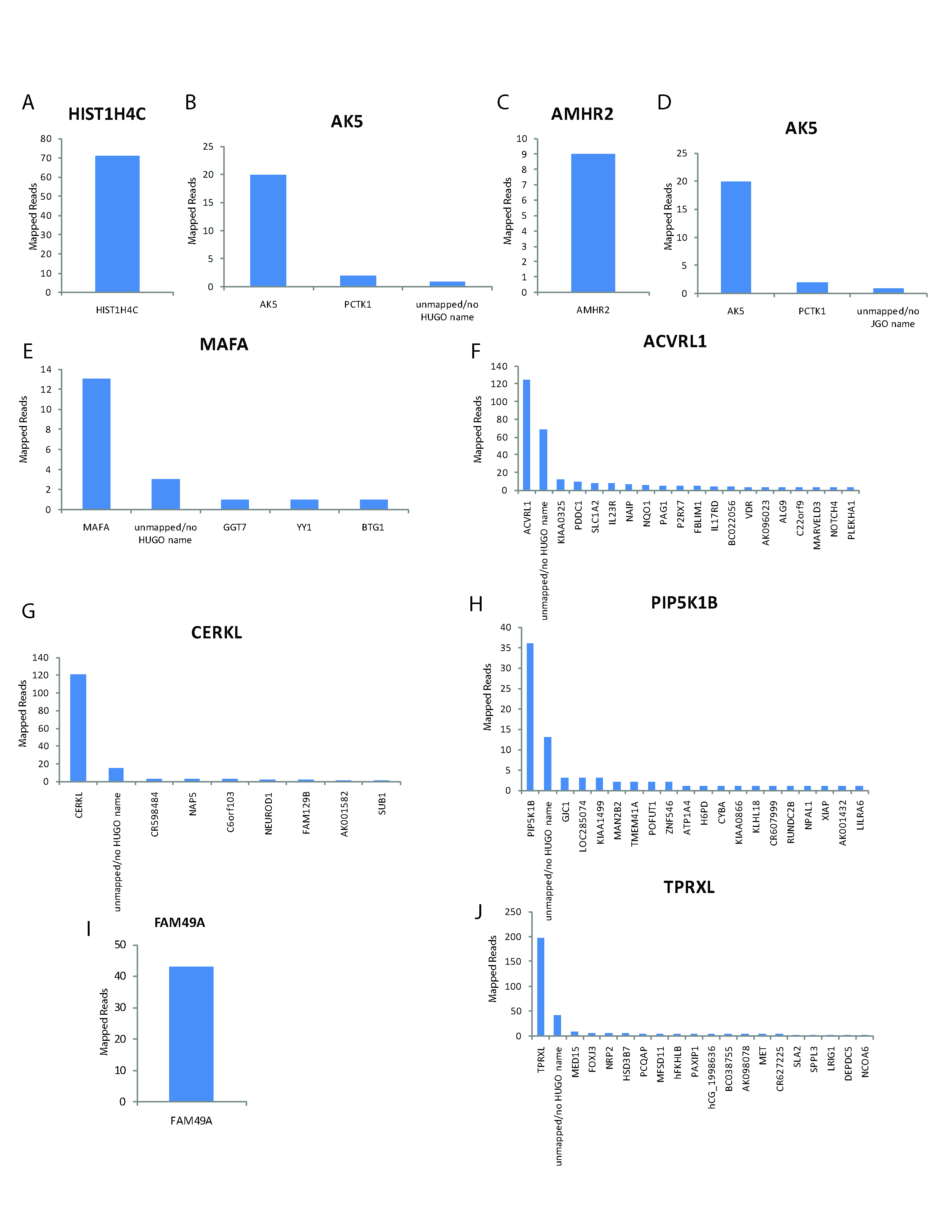

Supplement: Figure S14 — Alternate mappings for genes detected by SMS only in DU145. We analyzed alternate mappings for the reads attributable to each of the nine genes we observed to be detectable only by SMS in DU145 using reads from both replicates. In all nine cases, reads mapped most strongly to the genes of interest, suggesting that the detection of these genes is not an artifact of mismapping. The top 20 alternate mappings, ordered by mapping read count, are shown in the graph. (TIF) [file pone.0017305.s014.tif]

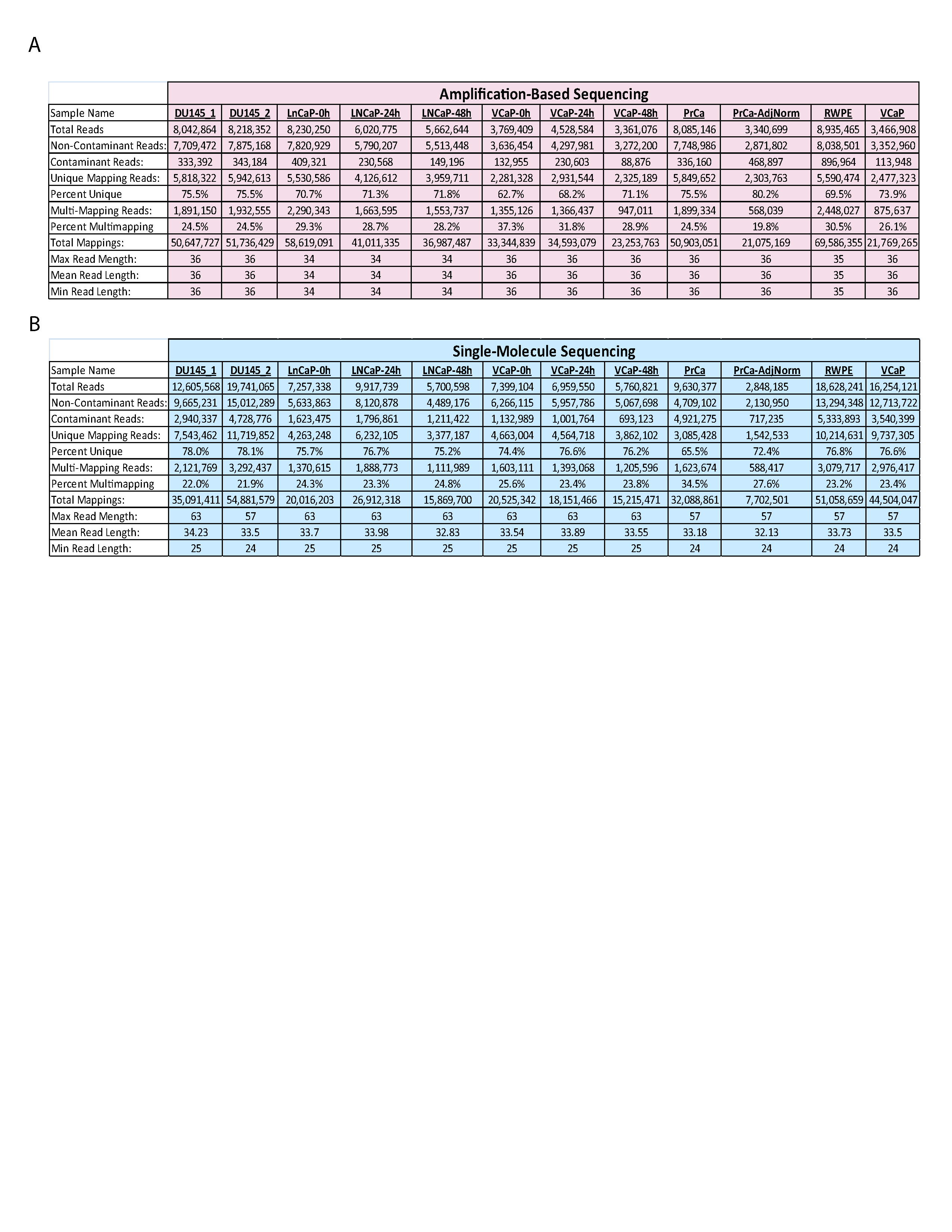

Supplement: Table S1 — Sample statistics in (A) amplification-based sequencing and (B) single-molecule sequencing technologies. (TIF) [file pone.0017305.s015.tif]

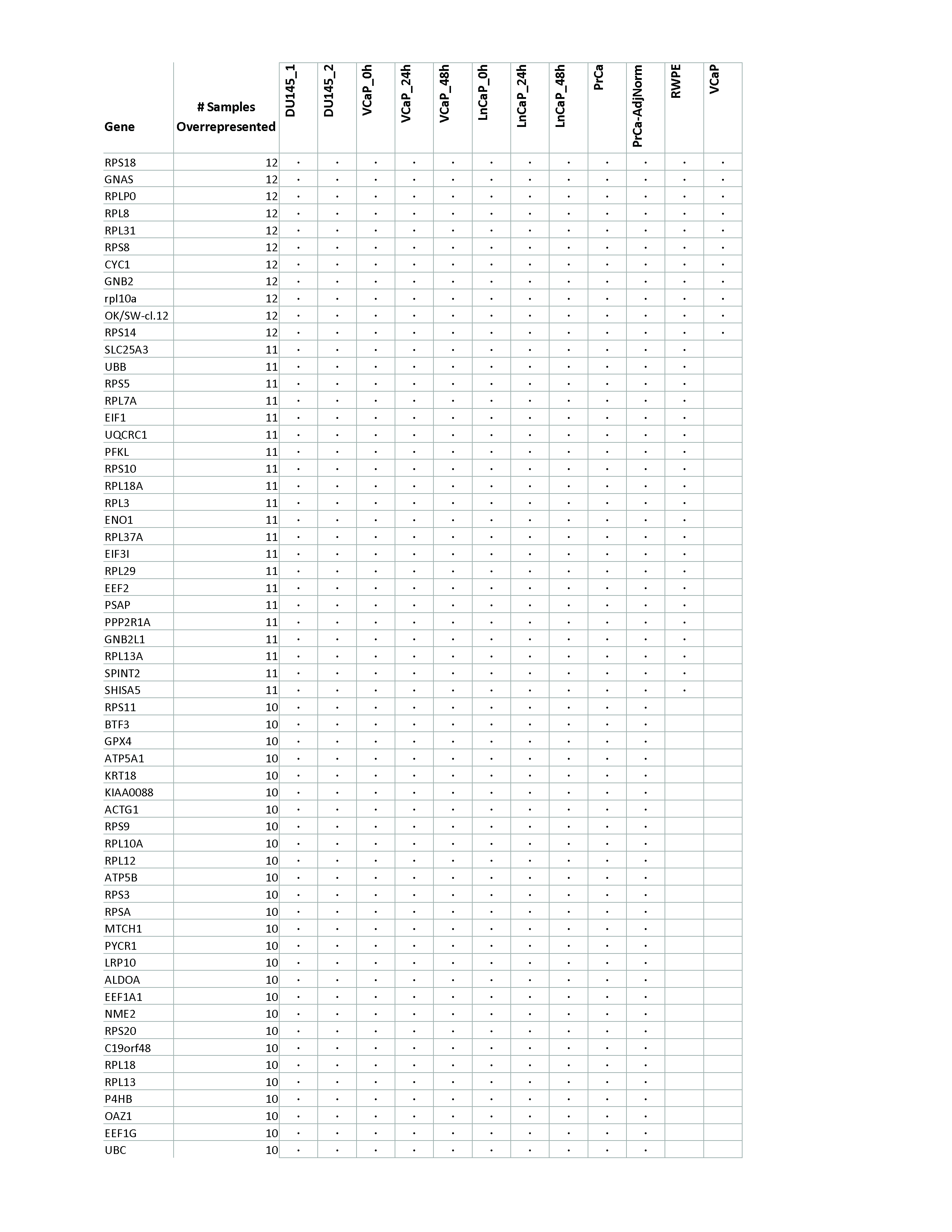

Supplement: Table S2 — Recurrently over-represented genes in amplification-based sequencing in ten or more samples. Of the 393 genes are recurrently within the top 500 over-represented genes by total read count in five (40%) or more samples, these 59 are seen most often, occurring in at least 10 samples. (TIF) [file pone.0017305.s016.tif]

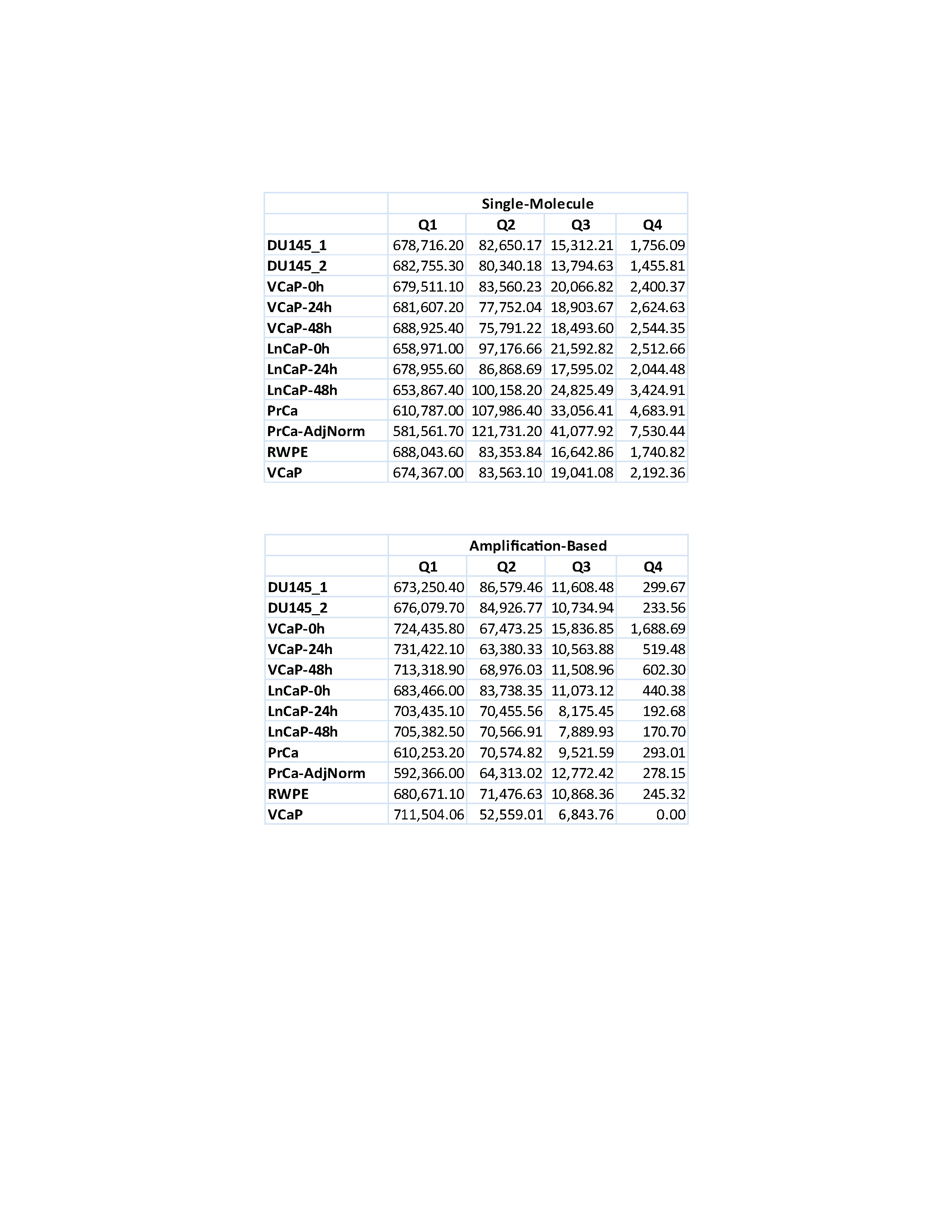

Supplement: Table S3 — Sum of normalized expression values per quartile by sample in AS and SMS. We observe that the number of reads aligning to transcripts seen in the third and fourth quartiles is consistently greater in SMS than AS across the sample set. (TIF) [file pone.0017305.s017.tif]

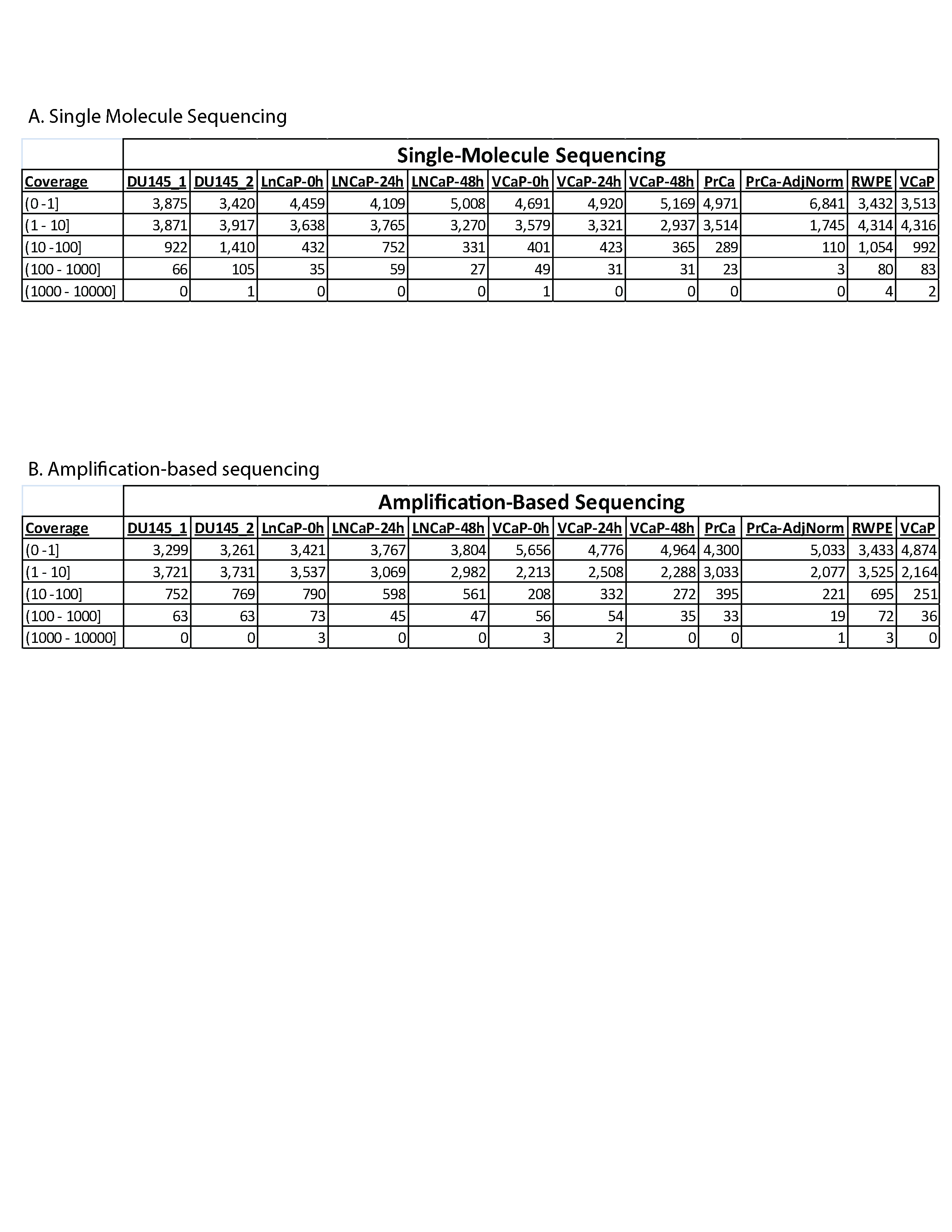

Supplement: Table S4 — Gene-level read coverage of observed transcripts. (A) and (B) illustrate the number of genes with coverage values at various depths in single molecule and amplification-based sequencing, respectively. (TIF) [file pone.0017305.s018.tif]

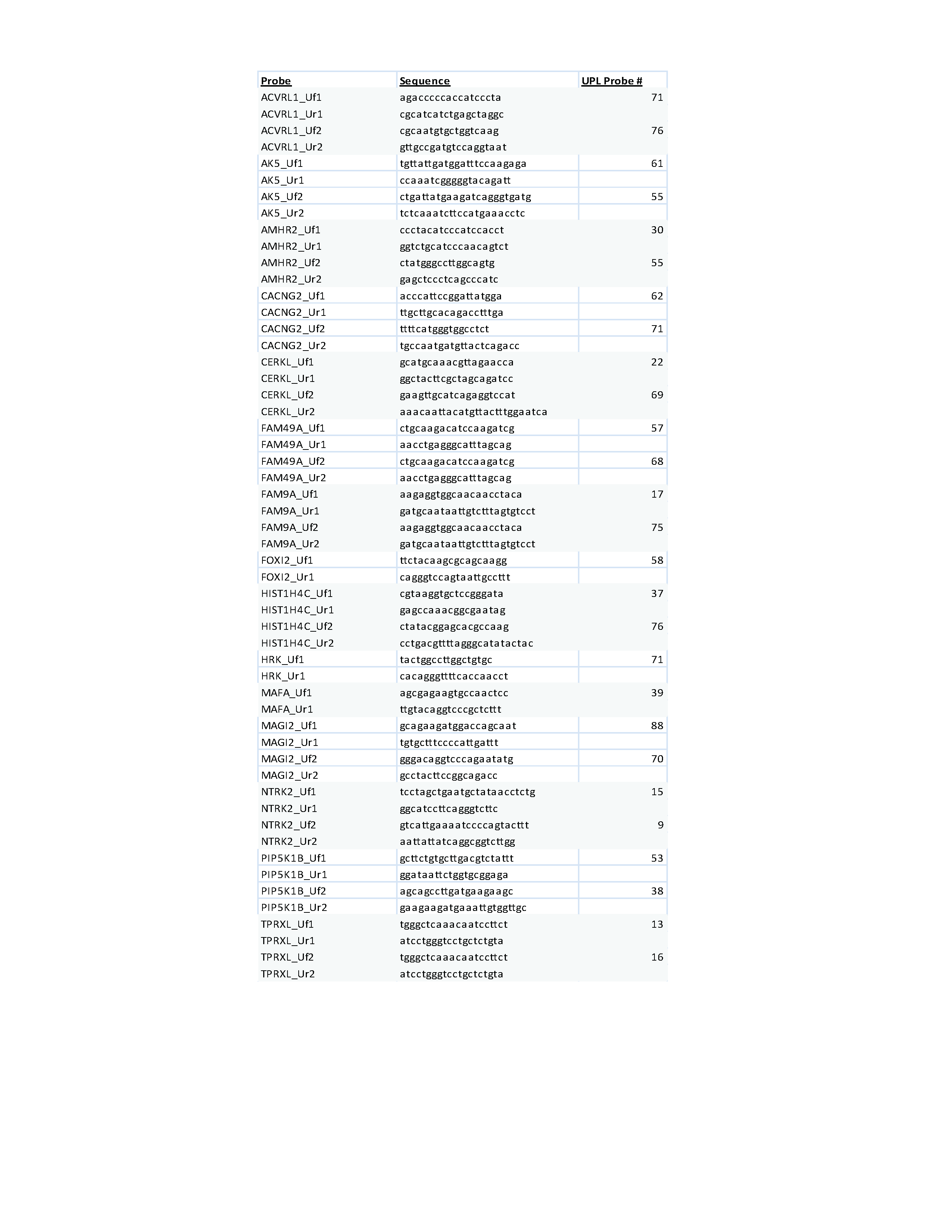

Supplement: Table S5 — Primers used for validating transcripts seen only by SMS. All experiments were performed in duplicate using two primer pairs per candidate gene when possible. (TIF) [file pone.0017305.s019.tif]
